# Perceptual learning in a non-human primate model of artificial vision

**DOI:** 10.1038/srep36329

**Published:** 2016-11-22

**Authors:** Nathaniel J. Killian, Milena Vurro, Sarah B. Keith, Margee J. Kyada, John S. Pezaris

**Affiliations:** 1 Department of Neurosurgery, Massachusetts General Hospital, Boston, MA 02114; 2 Department of Neurosurgery, Harvard Medical School, Boston, MA 02115.

## Abstract

Visual perceptual grouping, the process of forming global percepts from discrete elements, is experience-dependent. Here we show that the learning time course in an animal model of artificial vision is predicted primarily from the density of visual elements. Three naïve adult non-human primates were tasked with recognizing the letters of the Roman alphabet presented at variable size and visualized through patterns of discrete visual elements, specifically, simulated phosphenes mimicking a thalamic visual prosthesis. The animals viewed a spatially static letter using a gaze-contingent pattern and then chose, by gaze fixation, between a matching letter and a non-matching distractor. Months of learning were required for the animals to recognize letters using simulated phosphene vision. Learning rates increased in proportion to the mean density of the phosphenes in each pattern. Furthermore, skill acquisition transferred from trained to untrained patterns, not depending on the precise retinal layout of the simulated phosphenes. Taken together, the findings suggest that learning of perceptual grouping in a gaze-contingent visual prosthesis can be described simply by the density of visual activation.

Contemporary artificial vision devices are based on inducing small, discrete visual percepts, called phosphenes, generated with electrical stimulation^1^,^2^,^3^,^4^,^5^,^6^,^7^,^8^. Users of these devices face the task of grouping collections of discrete elements into perceptual wholes to recognize visual content. While perceptual grouping has often been studied with simple stimuli such as gratings or lines^9^,^10^, an artificial vision based paradigm presents an excellent opportunity to understand learning of perceptual grouping, with potentially important translational benefits. We previously created and tested a gaze-contingent thalamic visual prosthesis simulation in humans for both individual letter recognition^11^ and reading^12^. In these two studies we assessed behavioral letter recognition and reading performance as a function of font size and phosphene count in tasks that required well-developed skill in perceptual grouping. Results from those studies provide a reference for the possible visual acuity that can be achieved from a visual prosthesis with a given electrode count and thus act as a guide for visual prosthesis design. Interestingly, we also found that performance improvements can be seen within a single session, suggesting that learning is important for optimal visual prosthesis utility, and may have substantial impact on both device design and post-implant therapy. In order to study this learning in greater detail, and as preparation for electrophysiological work to be reported in a separate publication, we developed an animal model for visual prostheses where, after extensive training, animals are capable of performing similarly to humans on the letter recognition task^11^. Results from that training effort are reported here.

We used our artificial vision simulation to explore the behavioral correlates of learning a perceptual grouping skill that underlies visual prosthesis use, in naïve adult non-human primates. The ability of humans (and, as demonstrated here, of primates) to group visually-disconnected elements into a perceptual whole, an emergent behavior called perceptual grouping^13^,^14^, is thought to depend on processing in the visual system. The primary visual cortex is known to be an important structure in perceptual grouping^15^,^16^,^17^, and other visual areas have been implicated as well^18^,^19^. This processing is modulated by perceptual learning, goal-directed attention, and in general, visual experience^20^,^21^,^22^. Perceptual grouping has often been studied as a pre-existing skill^23^,^24^ or in terms of relatively short-term learning^21^,^22^, but we know little about the development of perceptual grouping skills over long periods of time (*i.e*. many months). We previously found that humans are capable of performing letter-based perceptual grouping tasks with no training^11^,^12^ and exhibited learning within a single session^11^. Others have observed perceptual learning in humans over a small number of days with trial counts on the order of thousands^22^,^25^. This ability to quickly reach high performance levels makes the study of long-term perceptual learning in humans difficult. Considering alternative animal models, naïve non-human primates have been shown to require many days to learn a relatively simple perceptual grouping task, even after consolidating task rules^21^. The potentially longer learning time course in non-human primates permits study of the development of perceptual grouping skills.

We specifically investigated the ability to group dynamic discrete visual elements into percepts of individual letters. Psychophysical testing using simulated artificial vision in sighted humans has previously been performed using stimuli, e.g. letters, viewed through patterns of simulated phosphenes^2^,^4^. Our experimental framework is based on psychophysical testing using simulated artificial vision in sighted humans that we have now extended to an animal model. In our paradigm, subjects first viewed spatially static letters through gaze-contingent patterns of simulated phosphenes and were then required to identify the letters by selecting from matching and distractor targets presented in clear view (Fig. 1)^11^. The targets in the choice phase were always presented in clear view and the cue letters were presented exclusively in clear view during the initial part of training. To distinguish between visual stimulation in the experiments and perception using phosphenes, we will refer to the presented view of a letter through simulated phosphenes as *phosphene view*, and perceiving objects through simulated phosphenes as *phosphene vision,* a new (to the subjects) visual modality. The phosphene view can be understood through Fig. 2 and Supplementary Movie 1. Here, by presenting stimuli through the phosphene view mechanism, we trained three rhesus macaques (*Macaca mulatta*) to use phosphene vision to perceive the underlying letter forms.

The animals learned to recognize the 26 uppercase letters of the Roman alphabet using a new font called Stelio (Supplementary Fig. 1) that was designed for this work as an extension of the Sloan testing set. For clarity, we will use the term *letter* to indicate the conceptual representation of a visual form in an alphabet, like the Platonic ideal of a letter, and *glyph* to indicate the rendering of a given letter from a given font at a given size for presentation to a subject. This latter term from typesetting and computer graphics is similar to *optotype* that is used in visual acuity testing. Letters from the Stelio font were presented at sizes corresponding to 5 visual acuity (VA) levels and with gaze-contingent, retinally-stabilized phosphene patterns. After months of training, the animals were able to perform at a level close to their human subject counterparts described in our previous study^11^.

Through behavioral analyses we have identified the predictors of static task performance as well as the correlates of long-term learning dynamics. In our analyses of task performance, we found that the stimulus parameters of letter size, *phosphene density* (the mean spacing of phosphenes as they form a pattern across the visual field), and cue-distractor contrast all predicted subject performance. Additionally, eye movement behaviors, specific to the goals of the task, were important factors in performance. Furthermore, learning, that is, the long-term progression of performance, depended strongly on phosphene density. Finally, after months of training, we tested phosphenes in retinotopic locations that were not previously used and found that acquired skills had transferred to these new visual field locations. This transfer appears to constrain the potential neural mechanisms underlying learning to those that are global in nature despite the spatial specificity of discrete islands or clusters of neurons (see next paragraph) activated by each phosphene during stimulus presentation. We note, however, that we have not directly observed neuronal activity and that our results are based entirely on psychophysics. Future studies will be necessary to improve our understanding of how the learning of perceptual grouping takes place in the normal visual system and furthermore in the context of artificial vision with an abnormal visual system.

The implications of using gaze-contingent visual stimuli are important to emphasize, primarily the stabilization of a given phosphene pattern on the retina. While the patterns were presented on a computer monitor, their location on the screen was adjusted on a frame-by-frame basis in real time to compensate for the instantaneously measured gaze position so that the image of each phosphene when projected on the retina was fixed relative to the retinal surface. As the animal looks around, each phosphene image on the retina changes only in illumination to reflect different aspects of the underlying visual objects being presented to the animal, as they are revealed as the animal looks around the screen. We might poetically say the pattern of phosphenes across the retina would appear to shimmer with gaze changes, but not to move about. As the remainder of the early visual system is retinotopic, a similar fixed constellation of locations would appear through the volume of the LGN, and again across the surface of each retinotopic cortical area. If one imagines observing their changes in activity, they would also appear to shimmer, but not to move relative to the retinotopic map of the area. Thus when we use the term clusters of activity, we are referring to the retinotopically fixed but temporally dynamic pattern of neural firing that is created by a given phosphene pattern in each visual area. Furthermore, we hypothesize that learning of perceptual grouping may be a result of plasticity among these clusters of activity that have been selectively co-activated during task performance.

## Results

Animals were trained with cue stimuli presented in clear view to learn the basic task and to develop their recognition for all 26 letters before the primary training with phosphene view cues was initiated. The animals completed 1,175 ± 713 trials per day overall (except where indicated, all statistics include *n* = 3 animals, and are given as mean with standard deviation), and required 17,669 ± 2,056 trials to achieve 80% correct on each of the 4 largest VA levels with clear view letters. Before introducing phosphene view cues (Fig. 2), the animals were performing near 100% (94, 81, and 91%) for each of the three animals averaged over all clear conditions (all letters and the last 50 trials for each of the four font sizes logMAR 1.0–1.9). Thus, the task rules were well-learned before learning of phosphene vision was introduced. Complete learning curves for clear view letters are shown in Fig. 3a. Clear view cues continued to be used during training on phosphene vision as positive controls. The performance on clear view letters improved through the end of training to 96%, 94%, and 95% for the three animals.

Learning of the phosphene vision skill progressed, but at a substantially slower rate. The animals required 112,167 ± 81,976 trials to reach at least 80% correct on the three largest VA levels with the *p*_*1*_ phosphene pattern and 116,241 ± 76,899 trials to reach 80% correct on the largest VA level with each of the *p*_*1*_, *p*_*2*_, and *p*_*3*_ patterns. Learning curves for all conditions and each animal are shown in Fig. 3. As additional, more difficult, phosphene view conditions were introduced at variable points later in time, performance at their introduction tended to be higher than would be expected by chance. For instance, the animals required 6,354 ± 6,343 trials to reach 80% correct on the largest VA level with pattern *p*_*1*_ and 3,107 ± 2,971 trials to reach 80% correct with pattern *p*_*3*_. This untrained improvement suggests that learning of phosphene vision with a given pattern density is sufficient to improve latent performance with other, less dense patterns even when these other patterns have phosphenes in different retinotopic locations. This transfer was later confirmed in one animal through testing with two sets of novel patterns.

Animals performed significantly above chance (*p* < 0.05; binomial test) on most conditions (Fig. 4a). With few exceptions, no animals reached performance above chance on extremely difficult conditions (125 phosphenes or 0.7 logMAR letters). The trained animals performed at a level similar to human subjects with *p*_*2*_, the densest pattern used in our earlier work^11^, and relatively poorer on more difficult conditions (Fig. 4b). Because of the similar performance levels, the animal model appears to be useful as a platform for tests leading to eventual implantation in humans. The humans tested in Bourkiza *et al*.^11^ were effectively experts in recognizing letters of the Roman alphabet so they may have been able to direct attention to key features that distinguish different letters. The predisposition to attending to key features may partially account for the human subjects’ ability to perform the task without training. Future examinations of the precise viewing patterns in monkeys and humans will be necessary to understand species-specific differences in the time taken to learn the task. Employing stimulus sets that are foreign to all subject groups may also help shed light on the origin of the performance differences on more difficult conditions and the longer learning time course in non-human primates.

In addition to the modulation of performance by VA level and pattern density described thus far, performance was also driven by properties of glyphs. In commonly used fonts, many pairs of letters of the Roman alphabet have a large amount of visual similarity, making it a challenging symbol set to learn for naïve animals that have never been trained to discriminate letters. Recognition performance may be worse between letters having a high level of similarity, even if the letters themselves have distinctive features. While normal human subjects (e.g. English-readers in Bourkiza *et al*.^11^, or Vurro *et al*.^12^) may have learned to identify key distinguishing features of Roman letters, we expected naïve monkeys to lack this perceptual skill. We applied information theory to assess the effects of letter distinctiveness and similarity on performance. The distinctiveness of individual letters was estimated using the information theoretic definition of entropy (*Η* ), which in this case quantifies the uncertainty associated with the pixel values (black or white, see Methods) of the glyphs used in the Cue phase. The similarity between letters was estimated using mutual information (*I* )^26^. Here, *I* quantifies the reduction in uncertainty of one arrangement of glyph pixels from knowledge of a second glyph’s set of pixels. Normalized mutual information (*Î* ) constrains the values to be between 0 and 1, with higher values of *Î* corresponding to higher similarity between glyphs. Because it is often useful to consider the contrast between cue and distractor glyphs, we established a dissimilarity index with value 1 − *Î*, which we will refer to as *cue-distractor contrast* (*Κ* ). These calculations are further described in the methods section, and distributions of *H* and *Κ* are given in Fig. 5a.

We analyzed performance as predicted by stimulus features using a generalized linear model (GLM) with the logit link function. We modeled task performance as a function of pattern density, *H*, and *K*. To avoid potential confounds due to learning, we used only the last 5,000 trials. In this subset of trials, there was no significant evolution in any of the variables (see Supplementary Figs 2 and 3). In Fig. 5b, performance is plotted as each GLM input parameter is varied along its range while the other parameters are held fixed at their mean value. When a parameter has a limited effect on predicted value, such as glyph entropy (*H* ), its curve will tend to lie near the global average performance, since the other parameters have been held fixed while the curve was generated. In contrast, when a parameter has a strong effect on predicted value, such as pattern density, the curve ranges from near chance at one end of the input parameter scale, to well above chance near the other end. When controlling for VA level and pattern density, performance was generally greater for cue-distractor pairs with high contrast *K*, or, equivalently, little in the way of common features between the two glyphs (Fig. 5b). Higher cue entropy *H* was also positively correlated with recognition performance, suggesting that glyphs having similar counts of black and white pixels are easier to recognize. The mean values of VA level, density, *K*, and *H* were approximately stable during learning (after an initial training period that concentrated on higher density patterns). The statistical significance of these predictors, however, gradually increased over time, likely reflecting the gradual improvements in performance accompanying learning (Supplementary Fig. 3).

We examined the role of eye movement properties in the Cue phase of the task using a GLM in the same manner in which we examined stimulus features. Specifically, we examined saccade rate, saccade amplitude, and microsaccadic jitter during fixation, all consistent with an underlying behavior of exploration. We hypothesized that higher saccade rates and microsaccadic jitter would correlate with increased performance. Increases in both behaviors should result in more of the glyph area being covered, allowing the animal to gather more information about the letter. The jitter value was taken to be the RMS radial eye position about the mean during each fixation period. Each trial was assessed with a single jitter value that was the mean of the RMS values over all fixations in the trial. Examining blocks of trials toward the end of data collection, we found that performance was overall greater when animals explored cue letters with smaller saccades or with higher saccade rates (Fig. 6, Supplementary Figs 4 and 5). Furthermore, jitter in the Cue phase was significantly correlated with performance in M_VG_ and M_CH_, and a similar trend was seen in M_ST_.

### Learning Phosphene Vision

We next turned to the perceptual underpinnings of learned letter recognition using phosphene vision. In the task, the individual phosphenes must be integrated by the visual system to form a global percept of a letter^15^. Despite training to high levels of performance with clear view letters, the overall training times required to develop mastery of phosphene vision were long, and times required for mastery of individual conditions increased as phosphene density decreased (Fig. 3b). To our knowledge, learning of perceptual grouping has not been studied both at a high temporal resolution and over such long time periods as in our experiment. This is perhaps because perceptual grouping skills are often considered to develop during infancy for humans^27^, or comparatively simpler tasks requiring shorter training periods have been used^21^. It is important to note, however, that human subjects tested on virtual reality simulations of visual prostheses have shown detectable improvements in performance with training. Humans show improvements in visual acuity across multiple sessions as tested with the Landolt C optotype^28^, and in word recognition performance at 15 degrees eccentricity over the course of a month^29^.

We hypothesized that the learning time course of perceptual grouping is related to the density of the phosphene patterns in terms of the mean distance to nearest neighbors. We found statistically significant positive correlations between learning time constants and the reciprocal of pattern density, or the mean free area surrounding each phosphene, for all animals (Fig. 7). We have described the results here in terms of a relationship between the time constant and inverse density, but it should be noted that this relationship is equivalent to a positive correlation between the learning rate (the reciprocal of the time constant) and density. Task difficulty is in general an important factor underlying learning rates. Difficulty on this task is determined largely by the combination of VA level and pattern density. In Fig. 7b, we have shown a slice through learning time constants based on pattern density. When we perform the same analysis driven by VA level, a similar relationship emerges, but one that does not have strong correlations that are consistent across animals. The two observations prompt us to suggest that for this particular set of circumstances, density is the primary factor determining learning rate. The density relationship appeared to be fundamental: when taking the best (or worst) 10% of trials in terms of the predictors identified as being significant predictors for all animals (Figs 5 and 6; any trials with at least one predictor value in the top/bottom 10^th^ percentile were taken), the normalized time constant versus inverse density relationships were practically indistinguishable (Supplementary Fig. 6). Thus the time course of learning in this task was determined by the combination of pattern density and the innate learning rate of each animal.

Although the high correlations found between density and learning curve parameters strongly suggest that density is the primary force that drives learning, it is interesting to consider how stimulus familiarity might affect learning. In particular, we considered the role of familiarity with specific pattern-glyph views and familiarity with phosphene pattern manipulation through eye movements. Learning was likely minimally influenced by familiarization with associations between pattern-glyph views and letters because performance levels were maintained with novel phosphene patterns (see next paragraph). Considering behavioral familiarity with the task, there were some changes in eye movement behaviors over time, but these changes were not consistent across animals and did not precisely correlate with the learning curve changes (Supplementary Fig. 5). Thus, the animals’ familiarity with the gaze-contingent aspects of the task through the development of eye movement strategies was not identified as a consistent predictor of the learning time course. Furthermore, if we consider the total number of stimulus exposures as a proxy for presumed familiarity, the number of exposures was nearly the same for each pattern density, thus effectively controlling for familiarity effects across conditions. Learning rates were not significantly correlated with the total number of stimulus exposures, except at one font size in one animal where there were sufficiently more high density pattern exposures to observe a trivial correlation between the learning time constants and the number of exposures. We verified that the correlations, which were computed using global trial counts, were not an artifact of interleaving trials by computing the time constants separately based on the number of exposures for each condition. Because conditions and exposures were interleaved during training, the time constant-inverse density correlations were essentially the same as when a global reference frame was used; correlation coefficients between time constants and inverse densities ranged from 0.95 to 1.00.

Generalization of phosphene vision to patterns with elements in novel retinotopic locations would imply that perceptual grouping can transfer to collections of elements at locations in the visual field that were not co-trained. Studies have typically reported a lack of any transfer to untrained locations unless subjects receive so-called *double training* with an additional higher-level task, such as learning both Vernier acuity and orientation discrimination^10^,^30^,^31^. The double training studies highlight complexities of perceptual learning, but unfortunately do not lend themselves to a straightforward mechanistic explanation. Here, we have a single task, but we have co-trained various combinations of retinotopic locations using centrally-weighted but randomly-generated phosphene patterns. The higher density patterns, *p*_*1*_ and *p*_*2*_, are sufficiently dense so that some neurons recruited with these patterns will also be recruited with less dense patterns. The lower density patterns, *p*_*3*_ through *p*_*6*_, however, are sufficiently sparse so that there is little overlap in phosphenes between any two. Because performance trained with higher density patterns transferred to novel lower density patterns when they were introduced, there may have been an underlying plasticity mechanism that depended on the total number of active neurons in an area, independent of their arrangement into distinct clusters of activity for each phosphene pattern. It is possible that there was memorization of specific letter-pattern associations, but this is unlikely given that Cue phase eye movements were found to play a critical role in performance (Fig. 6). It is also possible that skills in gaze-contingent manipulation of specific phosphene patterns affected the observation of the density relationship, but this is unlikely because the density correlations were still present when examining only strong or weak predictor values (Supplementary Fig. 6). We investigated these possibilities by testing one animal (M_VG_) with two new, independently generated sets of phosphene patterns with the same density profiles and phosphene counts. If either the memorization or gaze-manipulation scenarios were true, performance should have been lower with the new patterns. This animal performed generally as well as with the primary training patterns (Fig. 8). Performance was furthermore generally greater with the second novel pattern set (Novel 2) compared to the first novel pattern set (Novel 1). It is unlikely that a substantial amount of learning would have occurred for set Novel 1, and particularly unlikely for set Novel 2, as it was presented on only a single day. The changes with novel patterns are consistent with a generic improvement in perceptual grouping skills and a continuation of learning as expected by our analyses that predicted performance for all animals was not yet saturated. This finding confirms earlier observations of skill transfer from high density patterns to lower density ones during initial phases of training, suggesting that learned perceptual grouping with one set of phosphene patterns engaged a global mechanism that generalized to arbitrary retinotopic assemblies.

We also considered the choice of presenting stimuli with two levels of phosphene contrast (black and white), and whether reducing the number of contrasts to one would adversely affect performance. In one animal (M_VG_), after the primary training, we tested both black-only and white-only phosphenes, re-rendering our glyphs in either black or white, on gray squares that matched the background. We compared performance on the one-contrast conditions to performance on the primary training conditions, which used the full letter-and-surrounding-square stimuli at two levels of contrast (see Supplementary Fig. 7). Performance was approximately the same for both one-contrast stimuli and was close to performance with two-contrast stimuli on lower-density conditions. One-contrast performance was weaker than two-contrast performance on higher density conditions. These observations suggest that performance on this task depends on shape perception that is independent of phosphene color but is dependent on contrast information.

## Discussion

We first demonstrated that monkeys can learn to recognize the 26 letters of the Roman alphabet in clear view at a range of font sizes. The ability of monkeys to make visual discriminations between pairs of letters of the Roman alphabet has, to our knowledge, not previously been demonstrated. The capability of monkeys to perform this task is, however, not surprising given that monkeys have previously been trained to recognize arbitrary non-letter visually-presented objects^32^,^33^, make tactile-visual associations using letters^34^, and associate visually presented letters or arbitrary symbols with reward magnitude^35^. Here, after learning to recognize letters in clear view and thus consolidate task rules, the monkeys learned to recognize the same set of letters using patterns of simulated phosphenes acting as sets of discrete visual elements, where each element mimicked activation of a small cluster of neurons in the lateral geniculate nucleus. Although the monkeys quickly achieved near-perfect performance with letters presented in clear view, they required months of training with phosphene views to reach performance levels close to that of untrained humans. The animals reached 80% correct on the clear view symbols in an average of 30 days (logMAR 1.0–1.9). This learning rate is comparable to findings in Livingstone *et al*.^35^, where animals also reached approximately 80% correct when associating reward magnitude with 26 symbols after 30 days of training.

Once animals demonstrated competence on the task with cues in clear view, the experimental data for hundreds of thousands of phosphene view trials were collected. The results from an analysis of changes in performance over time demonstrated that learning of phosphene vision is likely to fundamentally depend on plasticity driven by the number of co-activated clusters of neurons. Finally, we demonstrated that, as expected from this simple concept of functional plasticity in neuronal populations, learning generalizes to untrained activation groups in the visual field. The generalization may result from prior strengthening of synaptic or other functional weights among locations due to involvement in grouping during training with higher-density patterns. Certain retinotopic locations may have been activated with some phosphene patterns but not others, e.g. compare the highest to the lowest density patterns, recalling that these patterns are retinally stabilized. Certain phosphene locations may have been shared between more than one training pattern. Hence, some phosphenes that occurred in the novel patterns may have been encountered during prior training and not, in themselves, been novel. Differences in experimental design relating specifically to pattern overlap may therefore reconcile our finding of transfer of performance to novel locations in the visual field with others’ findings of a lack of transfer^21^. Nevertheless, the important conclusion is that perceptual grouping likely depends not on a specific group of neurons, but upon a population mechanism whereby the same global percept can be achieved regardless of which low-level computational units are selected. This conclusion falls in line with a growing body of evidence highlighting the importance of modulation from higher-order visual areas in perceptual grouping performance^36^,^37^,^38^.

In our studies, the contrast was striking between the immediate ability of humans to perform the task in phosphene-view^11^ and the utter lack of similar ability in animals (despite having very good familiarity with the letter stimuli and task architecture) with an orders-of-magnitude slower learning rate. The vastly slower learning process allowed us to perform the detailed analysis reported here. We subsequently re-examined performance time courses observed in humans^11^ to determine if the same relationship is present between learning curves and density in humans. We were unable to fit learning curves to human subject performance on the easiest conditions where performance was already near 100% correct, but were able to perform the fit on data collected with the three lowest density patterns, thus supporting the usefulness of a model where learning is slower. When performing these learning curve fits, we found, on average, the same linear correlation between time constants and the inverse of pattern density as seen in monkeys (Supplementary Fig. 8; see Supplementary Fig. 9 for raw time constant values in both species). This finding suggests that a fundamental learning mechanism based on pattern density exists in multiple primate species regardless of the baseline performance and intrinsic learning rate.

Letter recognition performance depended on multiple identified factors: VA level (font size), phosphene count, cue-distractor contrast, and exploratory eye movement behaviors (saccade rate, saccade amplitude, and microsaccadic jitter). Performance improved as VA level and phosphene count increased, as expected since both factors ease the task by increasing the availability of information about the stimulus. During the Cue phase, increased saccade rate and decreased saccade amplitude (saccades over a relatively smaller region of the visual field) were correlated with improved performance. These two observations are consistent with the idealized goal to gather information about the cue letter, maximally exploring the glyph in the allotted time by making more saccades and by making saccades that maintain gaze location within the letter region. In turn, microsaccadic jitter may increase available information by shifting the pattern around the letter; jitter has been shown in other studies to optimize the statistics of the retinal image, by emphasizing higher frequency content such as the boundaries between black and white regions of the stimulus^39^. We found that jitter was significantly correlated with performance in two of the three animals. Thus, the usefulness of eye movement jitter in gathering letter information may be related to individualized patterns of eye movement behaviors. Future studies examining eye movement properties across multiple subjects will be necessary to understand how the fine details of viewing behaviors relate to performance. Learning, however, was best explained by activation density and intrinsic learning rates of each animal, rather than reflecting information content of the stimuli or learned eye movement behaviors.

We found that the amount of experience required to learn phosphene vision is best described by the reciprocal of phosphene pattern density (the mean clear area surrounding each phosphene), normalized by the fundamental learning rate of each animal (Fig. 7). Once the animals were trained, performance was maintained when novel phosphene patterns were introduced, suggesting that learning represents improvements in a generic perceptual grouping capability. The findings thus far support the idea of a learning mechanism that could be simply described by neuronal co-activation that increases functional weightings in a manner proportional to the density of neuronal tissue activation within the early visual system. In this simple description, the strengthening of these functional connections then results in proportionate increases in performance. The exact nature of these putative connections is unclear, but long-term potentiation and long-term depression have recently been observed in macaque primary visual cortex in a spike timing-dependent plasticity paradigm^40^. It is therefore possible that learning may be the result of changes in the strengths of synapses among neurons in V1, *i.e.*, through Hebbian learning among co-activated cells.

Learning may occur in higher order visual cortices as well as V1, but the density of activation in V1 may serve as an appropriate basis for understanding our results. More precisely, we are referring to the activation density in retinotopic space projected onto V1, or equivalently activation density over the cortical surface. The centrally-weighted phosphene patterns used in this study, would appear as a relatively even density of activation clusters across the surface. It is important to note firstly that these clusters will be fixed in retinotopic space as described above (see the final paragraph before the Results section), and secondly that the concept of learning being related to discrete clusters of cortical activation is confounded by orientation tuning, contrast tuning, and potentially other factors. We have assumed that the animals can group the discrete phosphenes into a shape by integrating distributed visual elements from the bulk of a letter or the edges of the letter. The animals learned to use patterns with average inter-phosphene distances from 0.28 to 1.72 degrees (see Table 1). These distances might yield orientation-tuned responses, based on known receptive field sizes in V1, suggesting that orientation-tuning in V1 may play a role in learning^41^. The effect of orientation tuning may be stronger along glyph borders where there is higher contrast and at higher pattern densities where spacing decreases. Thus we cannot disambiguate the effect on learning from increased neuronal response amplitude due to orientation tuning or contrast-mediated response gains from the effect of the number of neurons activated. Given the potential confounds of orientation and contrast tuning, future studies may benefit from modifying the symbol set to control the distributions of edge orientation and contrast.

Our observations suggest that denser phosphene resolution in visual space (and hence denser activation of the retina, LGN, and V1, and so on) is associated with faster changes in connectivity during learning. The rates of change in the functional weights, whether they reflect striate synaptic plasticity^42^,^43^ or plasticity in connections with higher order visual areas^44^,^45^, are predicted to be relatively greater for denser patterns, emphasizing that learning results from a population effect. The putative neuronal plasticity underlying perceptual grouping thus has a rate of change that could be described as a function of co-activated neuronal population size. This functional plasticity could be supported by simple temporal summation of co-activated neurons at the initial visual encoding stage or through re-entrant activity^46^. Furthermore, task learning likely also involves higher-order regions of the visual system that represent letter identity, which may have been altered through plasticity mechanisms during the initial clear view training period. These hypotheses could be tested through analysis of chronic recordings made throughout learning in synaptically or otherwise functionally connected neurons in visual areas. Selective activation of these visual areas using evoked or artificial phosphenes placed within their receptive fields may provide an appropriate stimulus to probe learning of perceptual grouping.

## Methods

Using a paradigm for testing perception of retinally-stabilized discrete visual elements intended to simulate artificial vision, we examined the longitudinal performance of a letter recognition task in a non-human primate model (*Macaca mulatta*) to elucidate learning of element grouping. The following subsections described the methodological details of the experiments.

### Animal preparation

Three adult male rhesus macaques (animals M_VG_, M_ST_, M_CH_) were surgically implanted with custom-machined titanium head-holding posts. The animals were trained to sit head-fixed in a primate chair (B & M Plastics, Inc.). The chair was placed such that the eyes were 43 cm from a 22-inch CRT monitor (Viewsonic P220f) in a shielded recording chamber (Crist Instrument Co.), with neutral gaze position near the center of the monitor. The research protocols were approved by the Massachusetts General Hospital Institutional Animal Care and Use Committee (IACUC) and were carried out in accordance with the NIH Guide for the Care and Use of Laboratory Animals.

### Behavioral task

**Figure 1:**
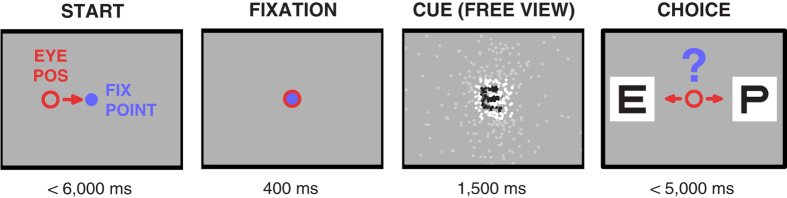
Behavioral task. After engagement (*Start phase*) and an initial fixation period (*Fixation*), animals are presented a gaze-contingent display of 1 of 26 cue letters filtered by a phosphene pattern (*Cue*). The animals are presented with a matching letter and a non-matching distractor, in the clear (*Choice*). Animals select between the two alternatives by fixation for an extended period, typically 500 ms, and receive a liquid reward if they select the choice target that matches the cue letter. The primary experimental variables were phosphene pattern (6 possibilities varying in total phosphene count) and letter size (5 possibilities matching logMAR 0.7, 1.0, 1.3, 1.6, 1.9). Additional variables that were typically integrated out by pooling were the cue letter (26 possibilities), distractor letter (25 possibilities), and matching target location (left or right).

The behavioral task followed a two-alternative forced-choice (2AFC) paradigm. Each trial began with an initial *Fixation phase* that required foveation of a small, circular target to ensure the animal was engaged with the task and looking at the center of the screen. After fixation (typically 300–800 msec), the *Cue phase* began where the animals could freely explore a cue glyph, presented either in clear view or phosphene view (*i.e.*, through one of the retinally-stabilized patterns of simulated phosphenes). Cue stimuli were shown for a fixed period of 1,500 msec. Animals began training with clear view stimuli having a 1,000 msec display time before transitioning to a 1,500 msec period for phosphene view training. Then came the *Choice phase* where animals had several seconds to select between two alternatives presented in the clear, a *matching target* that matched the cue glyph and a *distractor target* that was a non-matching letter presented at the same font size (Fig. 1, Supplementary Movie 1). After every 50–100 trials, an inter-trial phase to view natural scenes for 5–10 seconds was inserted to maintain motivation. Cue stimuli were drawn from conditions spanning 5 VA levels, 7 viewing conditions (6 phosphene patterns plus clear view), and 26 letters for a total of 910 possibilities. Choice stimuli were presented on the left and right parts of the screen in either order for matching and distractor glyphs, expanding the total pool to 45,500 conditions (5 × 7 × 26 × 25 × 2). Selections were made pseudo-randomly in a balanced fashion, although since the full condition set could not be explored in a single day, conditions were not typically repeated in a given session. During the Choice phase, animals were free to visually explore the two alternatives for up to 5 seconds, and indicated their choice by fixating for an extended period (typically 400–500 ms) on one of the targets. Animals received a large liquid reward for selecting the matching letter and a small reward for selecting the distractor. Sequential trials proceeded with independent and identically distributed conditions. On a block-to-block basis, the task difficulty level was manually adjusted to maintain overall performance within 60–80% correct by changing the proportion of easy versus hard conditions (phosphene patterns and VA levels; see Supplementary Fig. 3). Conditions that resulted in failures to engage or aborted trials were treated as if they had not been presented.

### Design of the font and phosphene patterns

The selection of letters for stimuli was driven by the larger line of inquiry in the laboratory, to enable comparisons across species, and, importantly, to have a ready conversion to the widely-understood metric of visual acuity in units of logMAR or Snellen notation. For consistency with standard optometric exam stimuli and to extend the size of the training symbol set, a custom font was created for this study based on the Sloan optotypes (see Supplementary Fig. 1). By using 26 letters, instead of the 10 in the Sloan set, the number of cue/distractor letter pairs was increased from 90 to 650, which was expected to promote generalization of the task rules by reducing the presentation of specific letter pairs, and eliminate undesirable statistical biases from the smaller stimulus set^11^.

Phosphene patterns were based on the expected outcome of uniformly sampling the volume of the LGN with stimulating electrodes based on parameters given by an LGN atlas^47^,^48^ as used in our previous reports^11^,^49^. With anatomically localized stimulation in humans, elicited percepts often resemble small points of light^50^. In the macaque LGN, stimulation coupled with monitoring of eye movements has suggested that phosphenes induced through LGN stimulation are spatially localized and appear as small points of light^51^. Phosphene patterns were constructed to have visual field sampling characteristics taken from the LGN, *i.e.* analogous relationships between size and eccentricity, and density and position in the visual field^12^. Patterns were denser toward the point of regard and tapered off toward the periphery, corresponding to the acuity profile across LGN. In the task, the animals were presented with the cue letter viewed through different phosphene patterns that varied in overall density (pattern parameters are given in Table 1) or through clear text unaltered by the phosphene simulation.

To verify that our phosphene density calculations yielded a reasonable approximation to activation density in V1, we performed a comparison of density-eccentricity relationships using data from macaque retina^52^, LGN^48^, and V1^53^. Given the available data, the LGN representational density (the number of cells that directly encode information per unit area in visual space) closely approximates the representational density in the visual cortex. Others have similarly concluded that the magnification factors in macaque retina and V1 are approximately the same^54^. Thus, even though the pattern densities were derived from studies of the LGN, no additional transformations were necessary to approximate the density of V1 activation. Moreover, as the phosphene patterns represented an even sampling of the thalamic volume (generating a centrally-weighted pattern in visual space due to foveal magnification), they therefore represented an even sampling of the cortical surface.

### Stimulus presentation

Visual stimuli were presented on a computer monitor in front of the animal. Custom software was created for stimulus display in addition to task control, and data collection^11^,^12^. The monitor had a display area of 40 by 30 cm that subtended 53 by 40 degrees in the visual field. Visual input was presented at a monitor resolution of 800 by 600 pixels and 120 or 160 Hz refresh rate. The eye position was sampled at 500 Hz, but update delays were set to 6.3 or 8 ms by the CRT monitor vertical refresh rates of 160 Hz and 120 Hz, respectively. Total per-frame processing time was typically 4 ms, including the 2 ms delay from the gaze tracker, with the rest of the video refresh cycle spent waiting for synchronization to vertical retrace. Latencies in the 6 to 8 ms range are comparable to state-of-the-art retinal stabilization systems (e.g. Rucci *et al*., Nature 2007 achieved a maximum latency of 10 msec with a similar stabilization system^39^) and are likely too short to be directly perceived by the animal.

Letters were rendered with black pixels inside a white square 10 degrees across. Carefully selected font sizes were used to render letters at the desired set of VA levels (logMAR 0.7, 1.0, 1.3, 1.6, and 1.9). To ease comparison with results from human subjects, these were the same VA levels used in our previous study^11^, although here all 26 letters were used for cue and distractors in all possible non-identical pairings, rather than the limited subset described in that report. Clear-view letters were presented in full detail as they would normally appear on the screen, without any phosphene-driven discontinuities and without gaze-contingent dynamics. Phosphene-view letters had a phosphene filter applied to the rendered letter in order to create the real-time animated stimuli, as discussed in the next section.

### Gaze-contingent (retinally-stabilized) cue display

**Figure 2:**
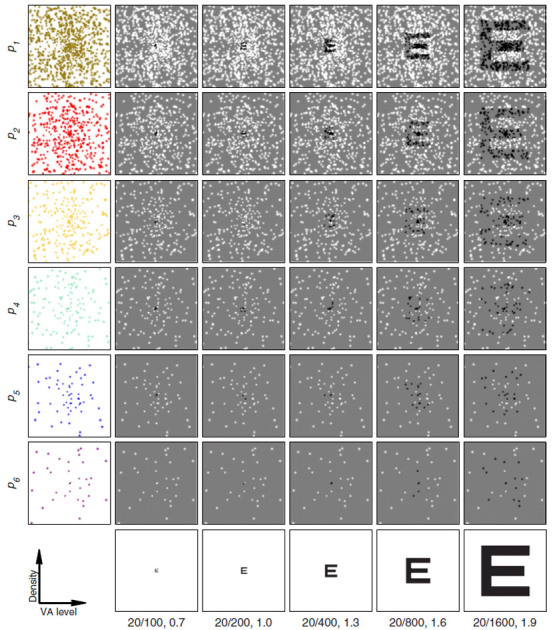
Static representations of simulated phosphene patterns. Here, the letter E is rendered with the gaze location at the center of the screen through the simulated phosphene patterns (rows 1–6 corresponding to phosphene patterns *p*_*1*_ to *p*_*6,*_ see Table 1 for description of nomenclature) and the VA levels tested (columns 2–6, Snellen and logMAR acuity values are listed at the bottom). Instantaneous gaze location is used to update the letter-pattern representation during each video refresh cycle to achieve retinal stabilization of the simulated phosphene pattern. For correct size at a normal reading distance of 40 cm, the image should be magnified such that the boxes bounding each plot have edges that are about 7 cm long and thus subtend about 10 degrees.

**Figure 3:**
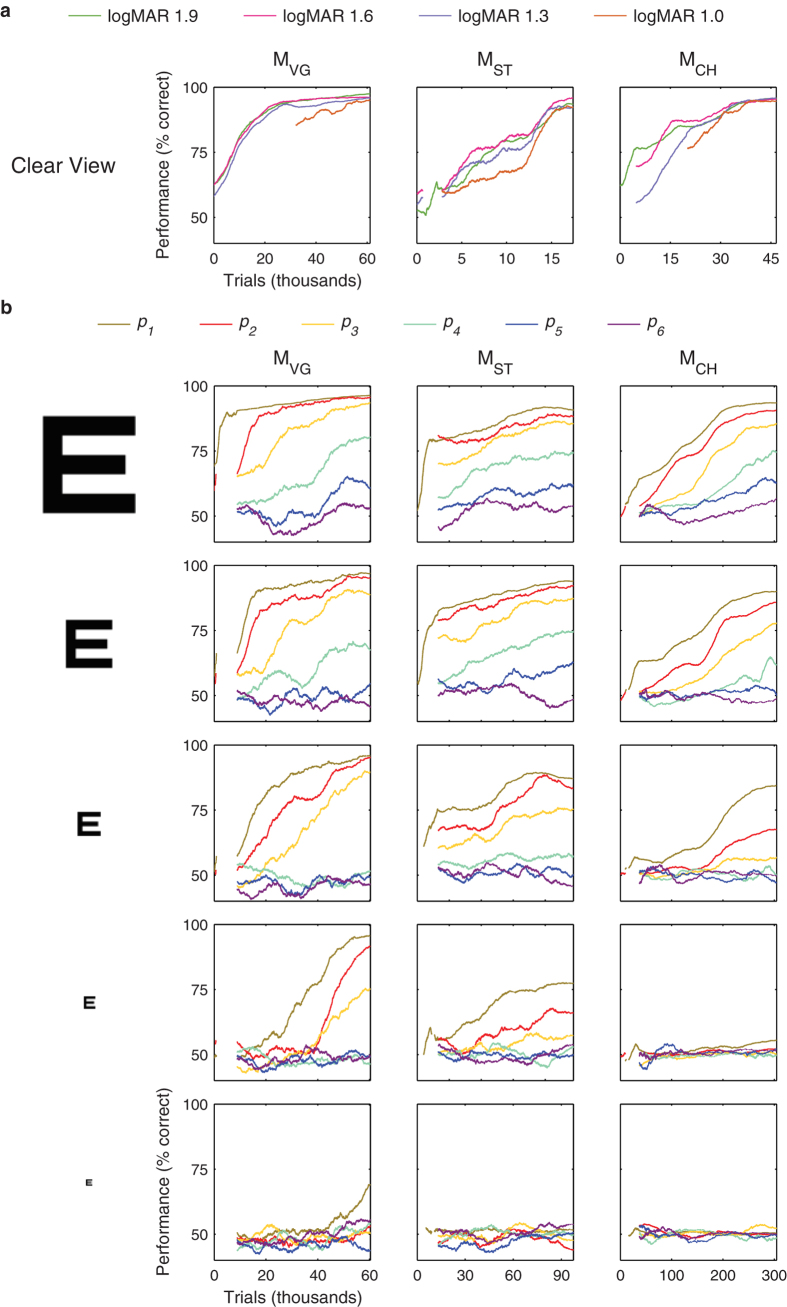
Learning of perceptual grouping. (**a**) Learning curves for clear text letters are plotted as performance versus total trials completed for each animal (columns, left to right: monkey M_VG_, M_ST_, and M_CH_). (**b**) Learning curves for all patterns at each VA level (first to last rows correspond to 1.9–0.7 logMAR in 0.3 logMAR steps). Learning progressed at different overall rates for each animal and depended on pattern densities (color scheme as in Fig. 2) and VA level (rows). Performance is shown with respect to a global start time. Gaps in the curves correspond to periods where a given condition was disabled.

**Figure 4:**
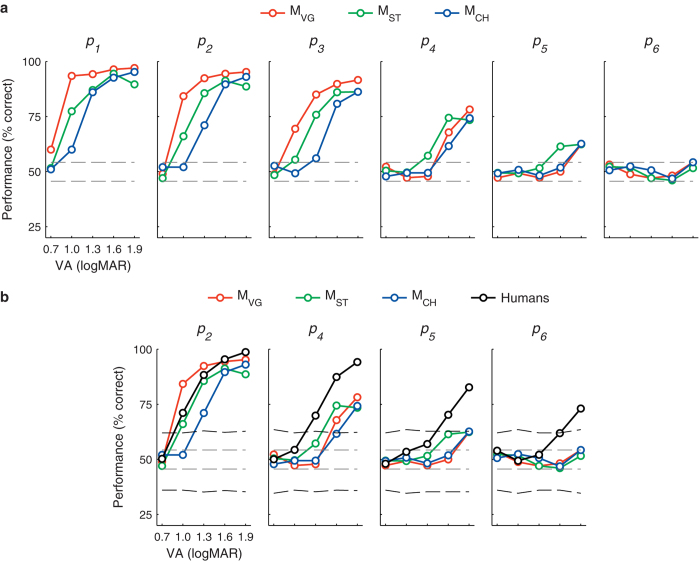
Letter recognition performance after learning. (**a**) Animals (red, M_VG_; green, M_ST_; blue, M_CH_) performed significantly above chance (above upper stippled line; *p* < 0.05, binomial test) on most conditions. Each data point represents the mean performance over the final 500 trials for the respective condition, spanning an average of 15 days. (**b**) Trained animals performed near human subject performance levels (black) with *p*_*2*_ and *p*_*4*_ patterns, but performance was relatively worse, compared to humans, on lower density patterns. Human curves were created from the previously reported population averages on the same task^11^. Because humans used patterns with slightly different phosphene densities, we compared to the conditions that were the most similar in terms of the number of phosphenes in the central 10-degrees. Central 10-degree phosphene counts for humans: *p*_*2*_, 370; *p*_*4*_, 110; *p*_*5*_, 45; *p*_*6*_, 25, compare to Table 1.

For Cue periods with phosphene view, a real-time stimulus was created using the gaze location to update the displayed image with each video refresh cycle. Overall, glyphs were rendered on a virtual surface centered on the screen and static in space; the phosphene pattern was relocated to track the instantaneous gaze location and thus revealed different parts of glyphs as the animal looked around the screen. Details of this method are given in previous reports^11^,^12^ and are summarized here. Gaze was tracked at a 500 Hz sampling rate with a video-based infrared eye tracking device (SensoMotoric Instruments, GmbH, model Hi-Speed Primate; accuracy: 0.25–0.5 degree, precision: <0.01 degree) using a pupil minus corneal reflection method, without temporal averaging. Gaze location was calibrated before each session, and typically updated every 1,000 trials during a session, using a 9-point calibration method^11^. The displayed image was constructed on a frame-by-frame basis from a black letter rendered on a 10 degree white square (surrounded by a 50% gray background) overlaid by the phosphene pattern translated to the gaze location. The intensity at each phosphene was the mean luminance below the phosphene’s location in the clear-view version of the glyph. The result was a retinally-stabilized pattern of simulated phosphenes controlled by the animals’ eye movements and used to explore image features for the duration of the Cue phase. Static representations of the letter *E* at the different VA levels viewed through the 6 simulated phosphene patterns are shown in Fig. 2 (dynamic versions that include the gaze-contingent nature of the phosphene view stimuli are seen in Supplementary Movie 1).

Because the reliability of generating particular colours of phosphenes has yet to be demonstrated, we chose to use stimuli for which performance could be directly compared to standard black-on-white optometric exam stimuli. The phosphene intensities were based on filtering glyphs with a set of 2-dimensional Gaussians, one at each phosphene location in the given pattern. We might expect that, in a visual prosthesis, stimulating on-center and off-center LGN cells might produce light and dark phosphenes, respectively. It is not necessary, however, that these precise color percepts be produced by the prosthesis; our assumption in the simulation is simply that the prosthesis can evoke two different categories of phosphenes.

### Animal training

Training on the task began with cue letters presented in clear-view so that the animals could learn the underlying paradigm and letter shapes, and progressed to more difficult conditions as their performance improved. A reduced set of letters and font sizes was found to aid initial training of the 2AFC format, but both were quickly expanded to the full set of conditions once the animals had grasped the underlying task. Once criterion performance of 80% correct on the four largest font sizes was obtained, phosphene views were enabled for the Cue phase. Typically, easier conditions (*i.e.* higher phosphene densities and larger letter sizes) were enabled first and more difficult conditions were enabled once performance improvements were achieved. Condition weights defining relative numbers of VA levels and phosphene patterns in the condition pool were manually adjusted during training to maintain overall performance between 60 and 80% correct and sustain motivation levels. Trials and days required to reach performance levels reported in Results were calculated by first smoothing performance with a Gaussian window having a 500-trial standard deviation for each condition and then identifying the overall trial number when performance crossed threshold.

### Eye movement analysis

Gaze location was parsed into saccade and fixation time periods in order to examine the role of eye movements in task performance. Saccade speed, amplitude, and rate (frequency of occurrence) were examined using saccade periods and microsaccadic jitter was examined during fixation periods. Gaze location was updated each video refresh cycle and resampled to a common rate of 160 Hz. To detect saccades, the Euclidean coordinates of gaze location were first separately filtered using a 10^th^ order 20 Hz low-pass finite impulse response filter and zero-phase digital filtering. Gaze location was not filtered in obtaining the other eye movement parameters. The filtered gaze signal was differentiated and thresholded at 25 degrees-per-second to define saccades. Fixation periods were defined from the end of one saccade to the beginning of the next. In the Cue phase, eye movements only within a radius of 5 degrees were considered. Parameters used as predictors were all taken as trial averages such that there was a single value for each trial for each predictor. The microsaccadic jitter values were calculated by taking the RMS value of the radial deviation from the mean gaze location for each fixation period, and then taking the arithmetic mean across all fixation periods in question.

### Letter information content

**Figure 5:**
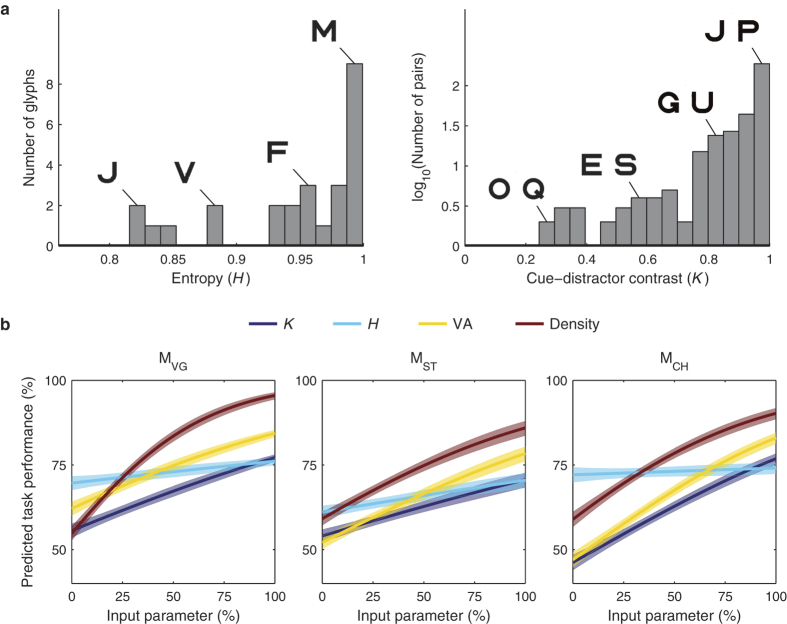
Performance is predicted by multiple stimulus variables. (**a**) *Left:* The distribution of glyph entropies is shown. Representative glyphs are called out as examples, including those with the lowest (J) and highest (M) entropy. *Right:* The distribution of cue-distractor contrast (*K* ) is shown, with representative examples identified, including the glyph pairs with the lowest (O and Q) and highest (J and P) contrast. (**b**) A GLM was used to fit performance with cue-distractor contrast (*K* ), glyph entropy (*H* ), VA level, and element density as input parameters. The vertical axes represent the task performance predicted by the GLM. Curves represent the predicted performance as each input parameter is varied along its range [0, 100%] and the remaining parameters are held at their mean values. Curves are coded by color according to which input parameter is being varied, and can thus be used to judge the relative impact of each parameter on the animal’s performance. Shaded regions represent 95% confidence intervals for the predictions. VA, density, and *K* were all significant predictors of performance (*p* < 0.05, chi-squared goodness of fit test). Glyph entropy *H* was also a significant predictor of performance for M_ST_ (*p* < 0.05) and a similar trend was seen for M_VG_ (*p* = 0.07). The analysis used the last 5,000 trials performed at logMAR 0.7 to 1.9 and phosphene patterns *p*_*1*_ to *p*_*6*_. The raw data used in the GLM are presented in Supplementary Fig. 2.

**Figure 6:**
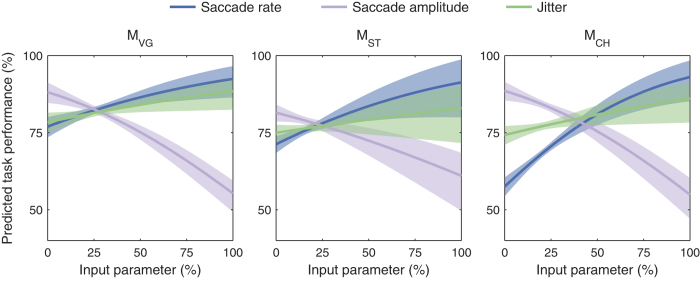
Effects of exploratory visual behaviors on performance. Performance was fitted with a generalized linear model using saccade rate (number of saccades per second), saccade amplitude, and microsaccadic jitter in the Cue phase as input parameters. As in Fig. 5, the horizontal axes represent the range for each input parameter normalized to [0, 100%], and the vertical axes represent the task performance predicted by the model. Each curve shows the influence of varying one input parameter while holding the other parameters fixed at their mean value. Shaded regions represent 95% confidence intervals for the predictions. *Correlation trends and significance of predictors:* Saccade rate and saccade amplitude were significant predictors of task performance in all animals and microsaccadic jitter was a significant predictor of performance in M_VG_ and M_CH_ (*p* < 0.05, chi-squared goodness of fit test) and a similar trend was seen in M_ST_ (*p* = 0.22). The analysis used the last 5,000 trials performed at logMAR 1.3 to 1.9 and phosphene patterns *p*_*1*_ to *p*_*5*_. The raw data used in the GLM are presented in Supplementary Fig. 4.

Estimates of the intrinsic information content of cue glyphs were made using entropy, *H*, see Equations 1–3 26. Here, each pixel within the globally circumscribing square outline for each VA level counted as one sample — white pixels outside of this square were not considered as these would bias the results. While spatial correlations, such as the strokes and curves of letters, carry additional self-information, these features were not taken into account. Instead, we made a simplifying decomposition of the 2-dimensional glyph pixel array into a 1-dimensional vector. Scrambling the pixel positions within the vector does not change entropy estimates because the calculation is performed assuming each pixel is independent. For black-and-white images, the maximum entropy is 1 bit, achieved by images with an equal number of black and white pixels. Most glyph images had a similar number of black and white pixels in the central glyph region, and thus tended to have entropy values near 1 (Fig. 5a). The relative information shared between cue and distractor glyphs was estimated using mutual information *I,* and *Î*, its normalized counterpart^26^. Normalized mutual information *Î* was computed using Equations 1–3, where *C* and *D* are the cue and distractor glyphs of interest, *p*(c) and *p*(*d* ) are the brightness probability distributions of the cue and distractor glyphs and *p*(*c, d*) is the joint cue-distractor brightness probability distribution. Glyphs were centered by construction, eliminating the need to additionally register the images for this analysis. All distributions were calculated with 2 brightness bins, corresponding to black and white pixels. Mutual information has no upper bound, but normalizing by the geometric mean of the image entropies guarantees that statistically independent images have *Î* = 0 and identical images have *Î* = 1.


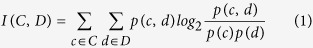



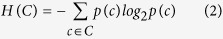



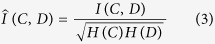


### Learning analysis: learning curves

**Figure 7:**
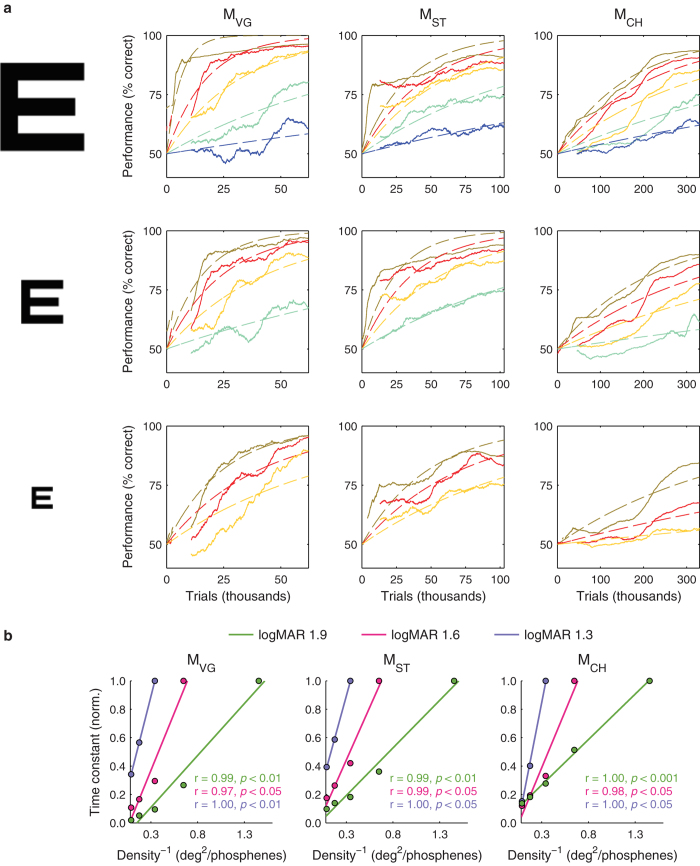
Learning of perceptual grouping depends on the density of visual elements. (**a**) Learning curves were fit with exponential functions (dashed lines) for the 3 highest VA levels and for phosphene patterns where performance went above the chance level for each animal. Trial counts are for the conditions plotted (the set of patterns at the given VA level). (**b**) Learning time constants, τ, obtained from the exponential fits increased linearly as a function of reciprocal density (the mean amount of clear space around each phosphene). The Pearson’s product-moment correlation coefficient and *p*-value are listed for each VA level in each animal.

**Figure 8:**
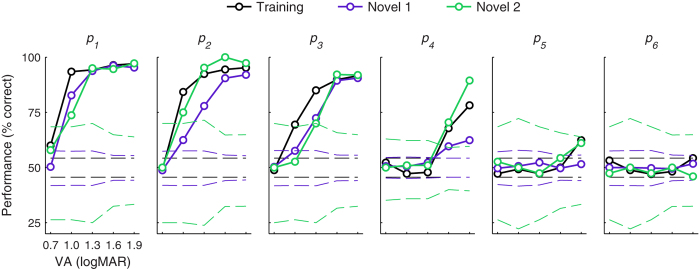
Learned perceptual grouping generalizes to untrained regions of the visual field. Performance with novel sets of phosphene patterns (purple, Novel 1; green, Novel 2) is compared here with performance on the original training patterns (black). Plot elements and conventions are the same as in Fig. 4a. Performance increased on some conditions with novel patterns, consistent with continuation of learning as each novel pattern was tested longitudinally. The last 500 trials (or fewer if less than 500 were completed) were included for each data point. Each size and pattern condition pair for the training stimuli had 500 trials over the last 17 days with the training set. Each condition pair for pattern set Novel 1 had 255 trials on average over 8 days of experiments. Each condition pair for pattern set Novel 2 had an average of 34 trials on the first and only day of experimentation with that set.

We estimated performance over time by first smoothing the binary trial outcome vectors for each condition with a Gaussian window having a standard deviation of 10% of the training time, chosen empirically to smooth out noise while retaining global features. Gaps in the curve for each condition, due to trial interleaving, were treated as missing values. Gaps less than 100 trials long were linearly interpolated for plotting and model fitting. The resulting *learning curves* were fitted with exponential functions from the first to the last trial for each pattern at each VA level of interest (*i.e.* the data presented in each set of axes in Fig. 7a). The exponential fits were defined by a learning time constant, *τ*_*l*_ , using a least-squares algorithm. By using time constants to describe the learning curves, the need for the animals to achieve any predefined performance level in order to perform a fit is eliminated. To avoid erroneous fits, however, only conditions where all animals achieved statistically significant performance above chance were included in the analysis. The time constants define the exponential fits to performance as a function of training time, *t*, and time interval, *P*(*t*), according to Equation 4. Training time is the amount of training, in our case it is the number of trials completed. The learning time constant, *τ*_*l*_ , is the number of trials required for performance to increase by 63% of the deficiency in performance with respect to 100% correct, at any time point during learning. It can be noted from Equation 4 that the learning time constant, *τ*_*l*_ , then conveniently gives the number of trials from the start of learning required to reach a performance level of 82% correct. The learning rate, which is the reciprocal of the time constant, can be thought of as the performance increase gained on each trial. For example, if the time constant is 10 trials and performance is at 50% correct, then the corresponding learning rate of 0.1 would give an average performance increase per trial of 3.2%. The learning curve model inherently accounts for saturation of performance, with performance ranging from 50% (background chance performance) to 100%. The model assumes that 100% correct performance would be achieved given enough time. A complete model of learning should include a description of forgetting, which is presumed to occur during breaks in training. Most of the experiments were collected with short intervals. The median experiment interval was 1 day for all animals (the minimum interval) and 90% of experiment intervals were 6 days or less. Because of these short inter-session intervals, we assumed that the effects of forgetting would not significantly impact our estimates of the overall learning time course.


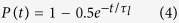


## Additional Information

**How to cite this article**: Killian, N. J. *et al*. Perceptual learning in a non-human primate model of artificial vision. *Sci. Rep.* **6**, 36329; doi: 10.1038/srep36329 (2016).

**Publisher’s note:** Springer Nature remains neutral with regard to jurisdictional claims in published maps and institutional affiliations.

## Supplementary Material

Supplementary Information

Supplementary audio

## Figures and Tables

**Table 1:** Simulated phosphene counts and densities.

Pattern name	Total phosphene count	Phosphene count in central 10°	Mean inter-phosphene distance in central 10° (deg)	Mean phosphene density in central 10° (phosphenes/deg^2^)
*p*_*1*_	4000	890	0.28	11.3
*p*_*2*_	2000	448	0.41	5.7
*p*_*3*_	1000	229	0.58	2.9
*p*_*4*_	500	121	0.83	1.5
*p*_*5*_	250	54	1.16	0.7
*p*_*6*_	125	29	1.72	0.4

This table lists, for each simulated phosphene pattern, the total numbers of simulated phosphenes, the phosphene count in the central 10 degrees of visual angle, the mean inter-phosphene distance in the central 10 degrees, and the mean density in the central 10 degrees. Contemporary retinal prosthesis devices include 16, 60, and 1500 phosphenes^8^,^55^. The experimental thalamic apparatus in our laboratory has 128 phosphenes, with an expectation of being able to expand that count by a factor of 2 or 4. With advanced materials, thousands of phosphenes should be possible^49^.

## References

[b1] Cai S., Fu L., Zhang H., Hu G. & Liang Z. Prosthetic visual acuity in irregular phosphene arrays under two down-sampling schemes: a simulation study. Conf. Proc. IEEE Eng. Med. Biol. Soc. 5, 5223–5226 (2005). 17281426 10.1109/IEMBS.2005.1615656

[b2] Cha K., Horch K. & Normann R. A. Simulation of a phosphene-based visual field: Visual acuity in a pixelized vision system. Ann. Biomed. Eng. 20, 439–449 (1992). 1510295 10.1007/BF02368135

[b3] Chen S. C., Hallum L. E., Lovell N. H. & Suaning G. J. Visual acuity measurement of prosthetic vision: a virtual-reality simulation study. J. Neural Eng. 2, S135–S145 (2005). 15876649 10.1088/1741-2560/2/1/015

[b4] Chen S. C., Suaning G. J., Morley J. W. & Lovell N. H. Simulating prosthetic vision: I. Visual models of phosphenes. Vision Res . 49, 1493–1506 (2009). 19504749 10.1016/j.visres.2009.02.003

[b5] Hayes J. S. . Visually Guided Performance of Simple Tasks Using Simulated Prosthetic Vision. Artif. Organs 27, 1016–1028 (2003). 14616520 10.1046/j.1525-1594.2003.07309.x

[b6] Dagnelie G., Barnett D., Humayun M. S. & Thompson R. W. Paragraph text reading using a pixelized prosthetic vision simulator: Parameter dependence and task learning in free-viewing conditions. Investig. Ophthalmol. Vis. Sci . 47, 1241–1250 (2006). 16505065 10.1167/iovs.05-0157

[b7] Ho A. C. . Long-Term Results from an Epiretinal Prosthesis to Restore Sight to the Blind. Ophthalmology 122, 1547–1554 (2015). 26162233 10.1016/j.ophtha.2015.04.032 PMC4516690

[b8] Stingl K. . Subretinal Visual Implant Alpha IMS–Clinical trial interim report. Vision Res . 111, 149–160 (2015). 25812924 10.1016/j.visres.2015.03.001

[b9] Li W. & Gilbert C. D. Global contour saliency and local colinear interactions. J. Neurophysiol. 88 (2002). 10.1152/jn.00289.2002 12424317

[b10] Hung S.-C. & Seitz A. R. Prolonged training at threshold promotes robust retinotopic specificity in perceptual learning. J. Neurosci. 34, 8423–8431 (2014). 24948798 10.1523/JNEUROSCI.0745-14.2014 PMC4061387

[b11] Bourkiza B., Vurro M., Jeffries A. & Pezaris J. S. Visual Acuity of Simulated Thalamic Visual Prostheses in Normally Sighted Humans. PLoS One 8, e73592 (2013). 24086286 10.1371/journal.pone.0073592 PMC3785446

[b12] Vurro M., Crowell A. M. & Pezaris J. S. Simulation of thalamic prosthetic vision: reading accuracy, speed, and acuity in sighted humans. Front. Hum. Neurosci . 8, 816 (2014). 25408641 10.3389/fnhum.2014.00816 PMC4219440

[b13] Kubovy M. The perceptual organization of dot lattices. Psychon. Bull. Rev . 1, 182–190 (1994). 24203469 10.3758/BF03200772

[b14] Roelfsema P. R. & Houtkamp R. Incremental grouping of image elements in vision. Atten. Percept. Psychophys. 73, 2542–2572 (2011). 21901573 10.3758/s13414-011-0200-0 PMC3222807

[b15] Roelfsema P. R. Cortical algorithms for perceptual grouping. Annu. Rev. Neurosci. 29, 203–227 (2006). 16776584 10.1146/annurev.neuro.29.051605.112939

[b16] Grossberg S., Mingolla E. & Ross W. D. Visual brain and visual perception: How does the cortex do perceptual grouping? Trends Neurosci . 20, 106–111 (1997). 9061863 10.1016/s0166-2236(96)01002-8

[b17] Kurylo D. D. Effects of visual cortex lesions on perceptual grouping in rats. Behav. Brain Res. 190, 182–188 (2008). 18395275 10.1016/j.bbr.2008.02.026

[b18] Yang T. & Maunsell J. H. R. The effect of perceptual learning on neuronal responses in monkey visual area V4. J. Neurosci. 24, 1617–1626 (2004). 14973244 10.1523/JNEUROSCI.4442-03.2004 PMC6730469

[b19] Petrov A. A., Dosher B. A. & Lu Z.-L. The dynamics of perceptual learning: an incremental reweighting model. Psychol. Rev. 112, 715–743 (2005). 16262466 10.1037/0033-295X.112.4.715

[b20] Ben-Av M. B., Sagi D. & Braun J. Visual attention and perceptual grouping. Percept. Psychophys. 52, 277–294 (1992). 1408639 10.3758/bf03209145

[b21] Li W., Piëch V. & Gilbert C. D. Learning to Link Visual Contours. Neuron 57, 442–451 (2008). 18255036 10.1016/j.neuron.2007.12.011 PMC2409109

[b22] Karni A. & Sagi D. The time course of learning a visual skill. Nature 365, 250–252 (1993). 8371779 10.1038/365250a0

[b23] Fang F., Kersten D. & Murray S. O. Perceptual grouping and inverse fMRI activity patterns in human visual cortex. J. Vis. 8, 2.1–9 (2008). 10.1167/8.7.2 19146235

[b24] Khoe W., Freeman E., Woldorff M. G. & Mangun G. R. Interactions between attention and perceptual grouping in human visual cortex. Brain Res . 1078, 101–111 (2006). 16500628 10.1016/j.brainres.2005.12.083

[b25] Kourtzi Z., Betts L. R., Sarkheil P. & Welchman A. E. Distributed neural plasticity for shape learning in the human visual cortex. PLoS Biol . 3, e204 (2005). 15934786 10.1371/journal.pbio.0030204 PMC1150289

[b26] Cover T. M. & Thomas J. A. Elements of Information Theory . (John Wiley & Sons, 2006).

[b27] Grossberg S. Linking the laminar circuits of visual cortex to visual perception: Development, grouping, and attention. in Neuroscience and Biobehavioral Reviews 25, 513–526 (2001). 11595271 10.1016/s0149-7634(01)00030-6

[b28] Chen S. C., Hallum L. E., Lovell N. H. & Suaning G. J. Learning prosthetic vision: a virtual-reality study. IEEE Trans. Neural Syst. Rehabil. Eng . 13, 249–255 (2005). 16200748 10.1109/TNSRE.2005.851771

[b29] Sommerhalder J. . Simulation of artificial vision: I. Eccentric reading of isolated words, and perceptual learning. Vision Res . 43, 269–283 (2003). 12535986 10.1016/s0042-6989(02)00481-9

[b30] Wang R., Zhang J.-Y., Klein S. A., Levi D. M. & Yu C. Vernier perceptual learning transfers to completely untrained retinal locations after double training: a ‘piggybacking’ effect. J. Vis. 14, 12 (2014). 10.1167/14.13.12 PMC4233766 25398974

[b31] Xiao L. Q. . Complete Transfer of Perceptual Learning across Retinal Locations Enabled by Double Training. Curr. Biol. 18, 1922–1926 (2008). 19062277 10.1016/j.cub.2008.10.030 PMC3045109

[b32] Lueschow A., Miller E. K. & Desimone R. Inferior temporal mechanisms for invariant object recognition. Cereb. Cortex 4, 523–531 (1994). 7833653 10.1093/cercor/4.5.523

[b33] Naya Y. & Suzuki W. A. Integrating what and when across the primate medial temporal lobe. Science 333, 773–776 (2011). 21817056 10.1126/science.1206773

[b34] Hsiao S. S., O’Shaughnessy D. M. & Johnson K. O. Effects of selective attention on spatial form processing in monkey primary and secondary somatosensory cortex. J. Neurophysiol. 70, 444–447 (1993). 8360721 10.1152/jn.1993.70.1.444

[b35] Livingstone M. S. . Symbol addition by monkeys provides evidence for normalized quantity coding. Proc. Natl. Acad. Sci. USA 111, 6822–6827 (2014). 24753600 10.1073/pnas.1404208111 PMC4020100

[b36] Gilbert C. D. & Li W. Top-down influences on visual processing. Nat. Rev. Neurosci. 14, 350–363 (2013). 23595013 10.1038/nrn3476 PMC3864796

[b37] Chen M. . Incremental Integration of Global Contours through Interplay between Visual Cortical Areas. Neuron 82, 682–694 (2014). 24811385 10.1016/j.neuron.2014.03.023

[b38] Yokoi I. & Komatsu H. Relationship between Neural Responses and Visual Grouping in the Monkey Parietal Cortex. J. Neurosci. 29, 13210–13221 (2009). 19846709 10.1523/JNEUROSCI.1995-09.2009 PMC6665213

[b39] Rucci M., Iovin R., Poletti M. & Santini F. Miniature eye movements enhance fine spatial detail. Nature 447, 851–854 (2007). 17568745 10.1038/nature05866

[b40] Huang S. . Associative Hebbian synaptic plasticity in primate visual cortex. J. Neurosci. 34, 7575–7579 (2014). 24872561 10.1523/JNEUROSCI.0983-14.2014 PMC4035519

[b41] Durand J.-B., Girard P., Barone P., Bullier J. & Nowak L. G. Effects of contrast and contrast adaptation on static receptive field features in macaque area V1. J. Neurophysiol. 108, 2033–2050 (2012). 22815398 10.1152/jn.00936.2011

[b42] Löwel S. & Singer W. Selection of intrinsic horizontal connections in the visual cortex by correlated neuronal activity. Science 255, 209–212 (1992). 1372754 10.1126/science.1372754

[b43] Rioult-Pedotti M. S., Friedman D., Hess G. & Donoghue J. P. Strengthening of horizontal cortical connections following skill learning. Nat. Neurosci. 1, 230–234 (1998). 10195148 10.1038/678

[b44] Han S., Jiang Y., Mao L., Humphreys G. W. & Gu H. Attentional modulation of perceptual grouping in human visual cortex: Functional MRI studies. Hum. Brain Mapp. 25, 424–432 (2005). 15852379 10.1002/hbm.20119 PMC6871716

[b45] Murray S. O., Schrater P. & Kersten D. Perceptual grouping and the interactions between visual cortical areas. Neural Networks 17, 695–705 (2004). 15288893 10.1016/j.neunet.2004.03.010

[b46] Sporns O., Tononi G. & Edelman G. M. Modeling perceptual grouping and figure-ground segregation by means of active reentrant connections. Proc. Natl. Acad. Sci . 88, 129–133 (1991). 1986358 10.1073/pnas.88.1.129 PMC50763

[b47] Malpeli J. G., Lee D. & Baker F. H. Laminar and retinotopic organization of the macaque lateral geniculate nucleus: Magnocellular and parvocellular magnification functions. J. Comp. Neurol. 375, 363–377 (1996). 8915836 10.1002/(SICI)1096-9861(19961118)375:3<363::AID-CNE2>3.0.CO;2-0

[b48] Erwin E., Baker F. H., Busen W. F. & Malpeli J. G. Relationship between laminar topology and retinotopy in the rhesus lateral geniculate nucleus: Results from a functional atlas. J. Comp. Neurol. 407, 92–102 (1999). 10213190

[b49] Pezaris J. S. & Reid R. C. Simulations of electrode placement for a thalamic visual prosthesis. IEEE Trans. Biomed. Eng. 56, 172–178 (2009). 19224730 10.1109/TBME.2008.2005973 PMC5446893

[b50] Brindley G. & Lewin W. The Sensations Produced By Electrical Stimulation of the Visual Cortex. J. Physiol . 196, 479–493 (1968). 4871047 10.1113/jphysiol.1968.sp008519 PMC1351724

[b51] Pezaris J. S. & Reid R. C. Demonstration of artificial visual percepts generated through thalamic microstimulation. Proc. Natl. Acad. Sci. USA 104, 7670–7675 (2007). 17452646 10.1073/pnas.0608563104 PMC1863473

[b52] Rolls E. T. & Cowey A. Topography of the retina and striate cortex and its relationship to visual acuity in rhesus monkeys and squirrel monkeys. Exp. Brain Res. 10, 298–310 (1970). 4986000 10.1007/BF00235053

[b53] Daniel P. M. & Whitteridge D. The representation of the visual field on the cerebral cortex in monkeys. J. Physiol . 159, 203–221 (1961). 13883391 10.1113/jphysiol.1961.sp006803 PMC1359500

[b54] Wassle H., Grunert U., Rohrenbeck J. & Boycott B. B. Retinal ganglion cell density and cortical magnification factor in the primate. Vision Res . 30, 1897–1911 (1990). 2288097 10.1016/0042-6989(90)90166-i

[b55] Ahuja A. K. . Blind subjects implanted with the Argus II retinal prosthesis are able to improve performance in a spatial-motor task. Br. J. Ophthalmol. 95, 539–543 (2011). 20881025 10.1136/bjo.2010.179622 PMC3345188

