# Supplementary material for: Perceptual learning in a non-human primate model of artificial vision
Source: Sci Rep. 2016 Nov 22;6:36329. doi: 10.1038/srep36329 (PMC5118870; doi:10.1038/srep36329)
Supplement: Supplementary Information [file srep36329-s1.pdf]

SUPPLEMENTARY INFORMATION

Perceptual learning in a non-human primate model of artificial vision

Nathaniel J. Killian[1,2], Milena Vurro[1,2], Sarah B. Keith[1], Margee J. Kyada[1], John S. Pezaris[1,2]

[1]Department of Neurosurgery, Massachusetts General Hospital, Boston, MA 02114
[2]Department of Neurosurgery, Harvard Medical School, Boston, MA 02115
nkillian@mgh.harvard.edu, john@pezaris.com

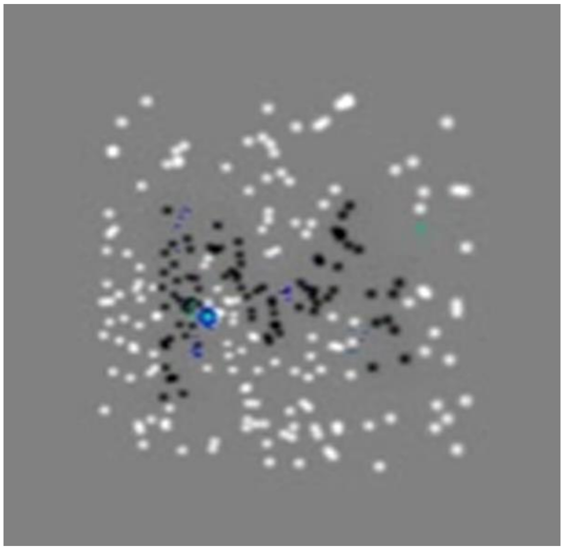

*Sample frame, cue phase exploration of letter **M**, from supplementary-movie-1.avi*

Supplementary Movie 1 | Recreation of animal's display. This movie is a recreation of one animal's display for an example 30 trials. The animal first explores the cue glyph with a randomly-selected phosphene pattern. The movements in the patterns are a result of the animal's use of eye movements to explore the glyphs. The gaze location, tracked with an infrared camera, is represented by the blue-green spots (in the video only, the animals did not see any markers representing their gaze location when performing the task). After exploring the cue, the animal then chooses between a letter matching the cue and a non-matching distractor. Successful trials are rewarded while the letter remains on the screen.

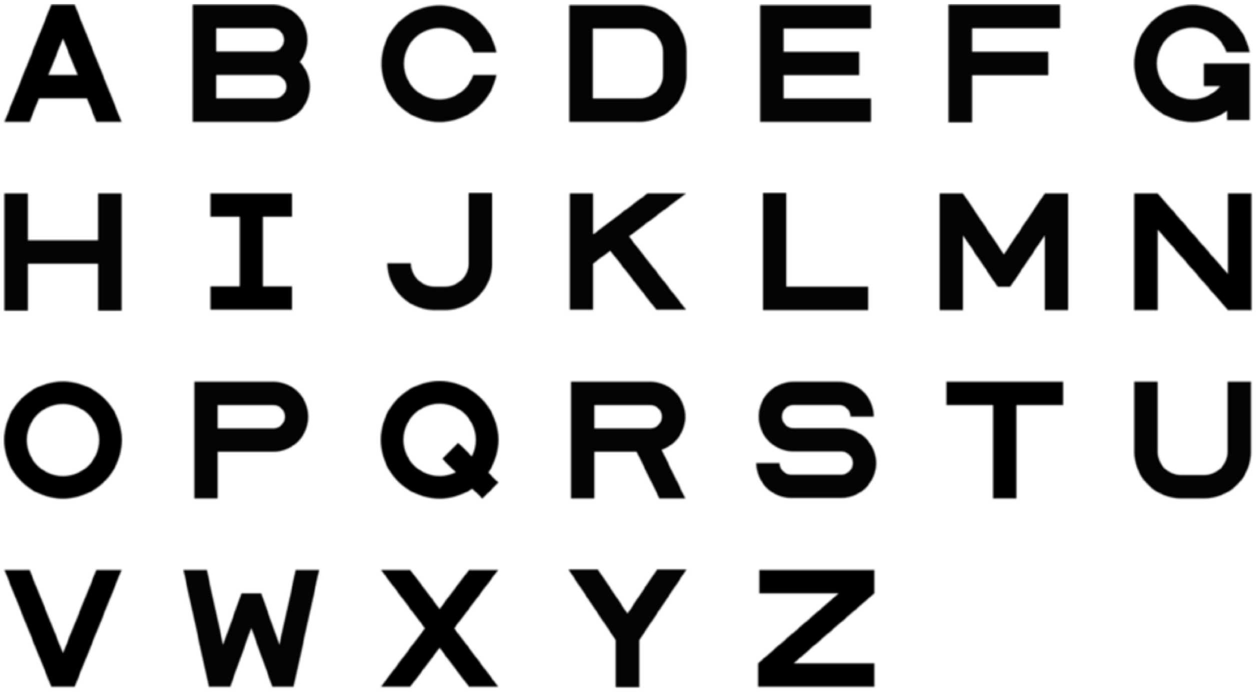

Supplementary Figure 1 | Glyphs in the Stelio font. An important part of traditional visual acuity tests is the implicit size of the pool of letters from which the presented stimuli are drawn, as the size of the pool affects the statistical analysis of the responses. For standardized tests administered in English speaking countries, such as the familiar Snellen chart or the more clinically common EDTRS test[1], the pool is the full set of 26 upper case letters, although only 9 letters appear in the Snellen chart, and 10 in the EDTRS set[2]. Because of this need to create an appropriate basis for recognition testing, we developed an extension of the ten Sloan letters[3] that comprise the 10 optotypes used in the EDTRS chart, creating a font with a full set of 26 uppercase letters. The resulting Stelio font was designed in cooperation with a font foundry (International TypeFounders, Inc.) to appear as normal as possible, and with high readability, in order to overcome some of the problematic aspects of earlier, similar efforts[4].

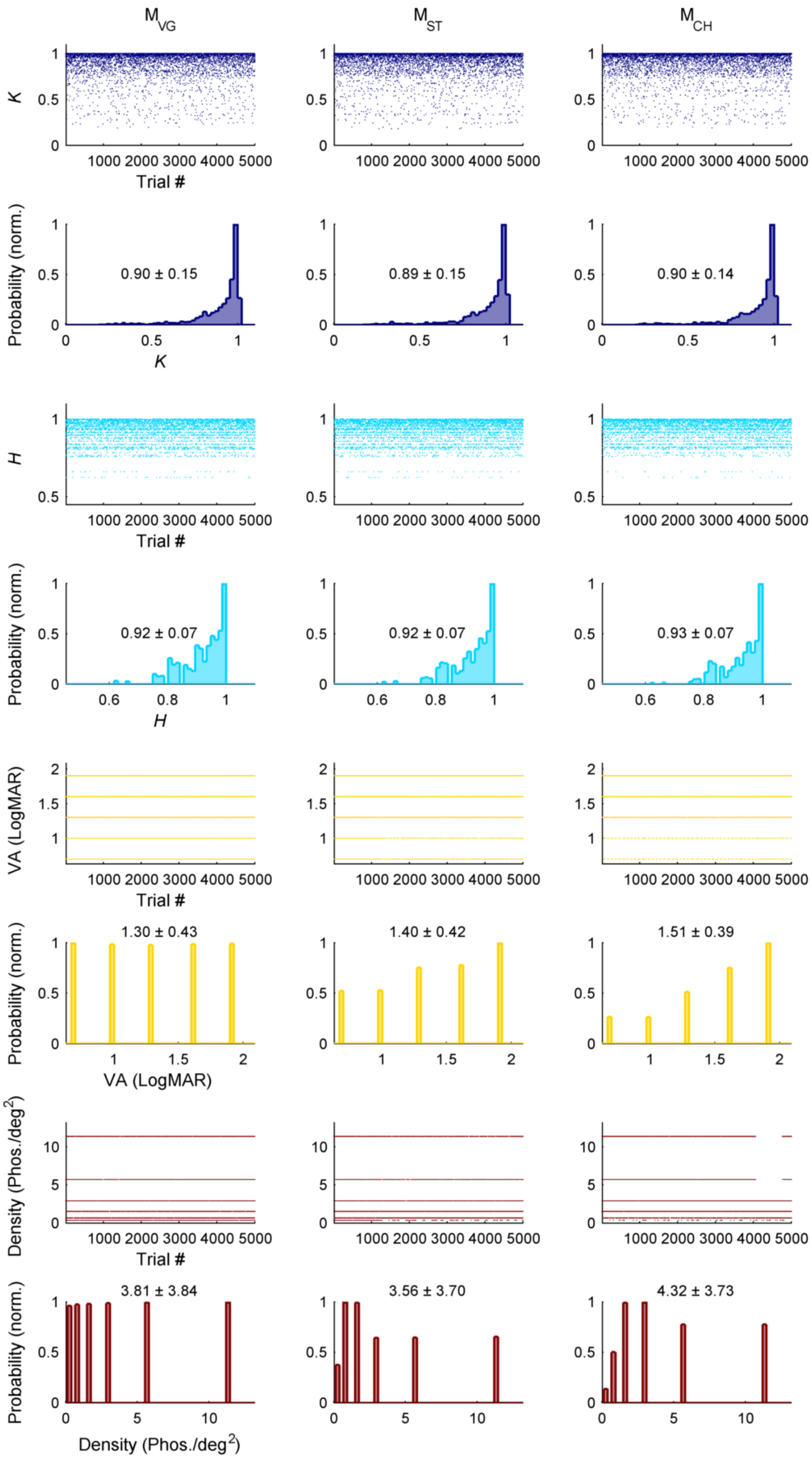

Supplementary Figure 2 | Distributions of stimulus predictors used in the GLM. The values for each stimulus predictor for each of the last 5,000 trials, which were used in the GLM procedure (see also Figure 5b), are plotted for each animal (columns). Predictors and units are: *K*, cue-distractor contrast; *H*, entropy; VA level, logMAR; and density (phosphenes/deg^2). Colors following from Figure 5b of the main text. The histograms for the predictor values are plotted normalized to the maximum probability of occurrence. The mean and standard deviation are given for each distribution.

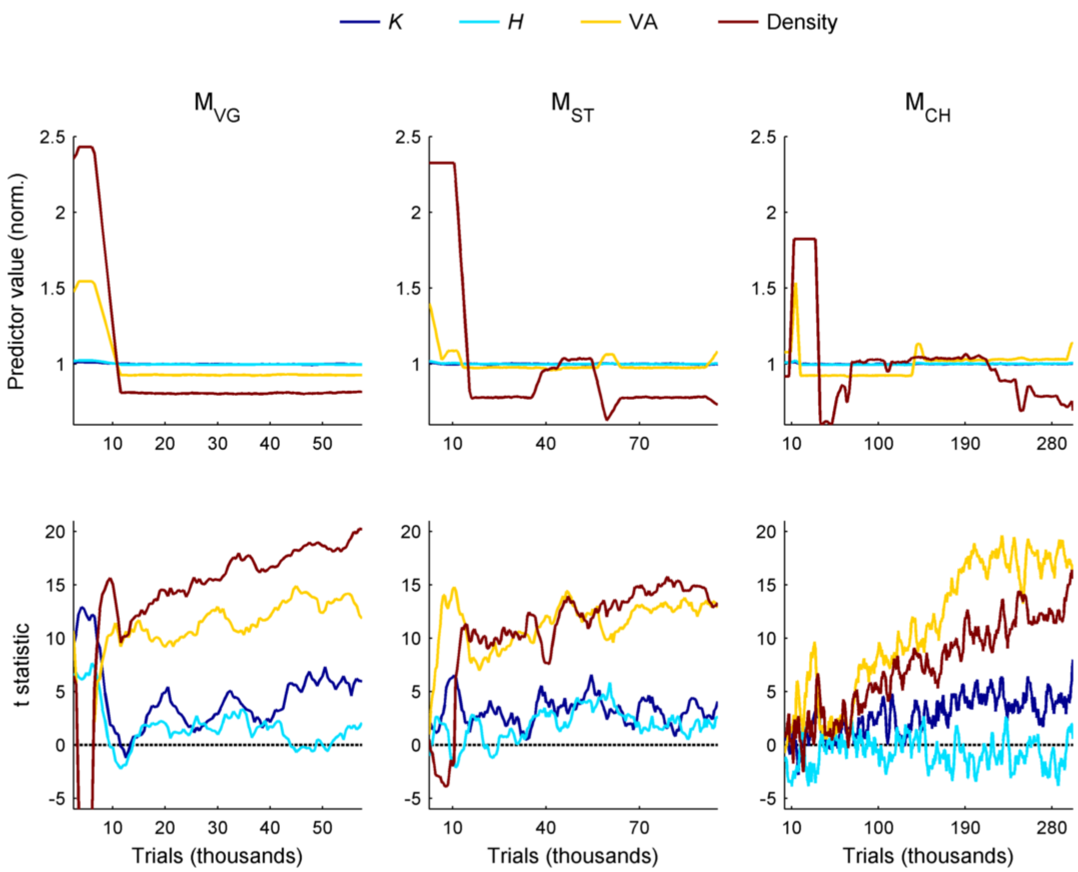

Supplementary Figure 3 | Time course of stimulus predictors. *Top row:* The time course is shown for each predictor value (relative to the global arithmetic mean value for each predictor; norm., normalized units). The jumps in density and VA level are from adjustments made during learning to help maintain performance between 60 and 80% correct (see main text). Predictor labels and colors are the same as those used in Figure 5b. Predictors *K* and *H* were approximately stationary throughout training. *Bottom row:* The time course is shown for the *t*-statistic of the coefficient for each predictor in the GLM described in the main text (Figure 5). Sliding averages were taken with a rectangular window to create the time courses in both panels (5,000 trials, 90% overlap; VA 0.7 to 1.9 and patterns p_1 to p_6).

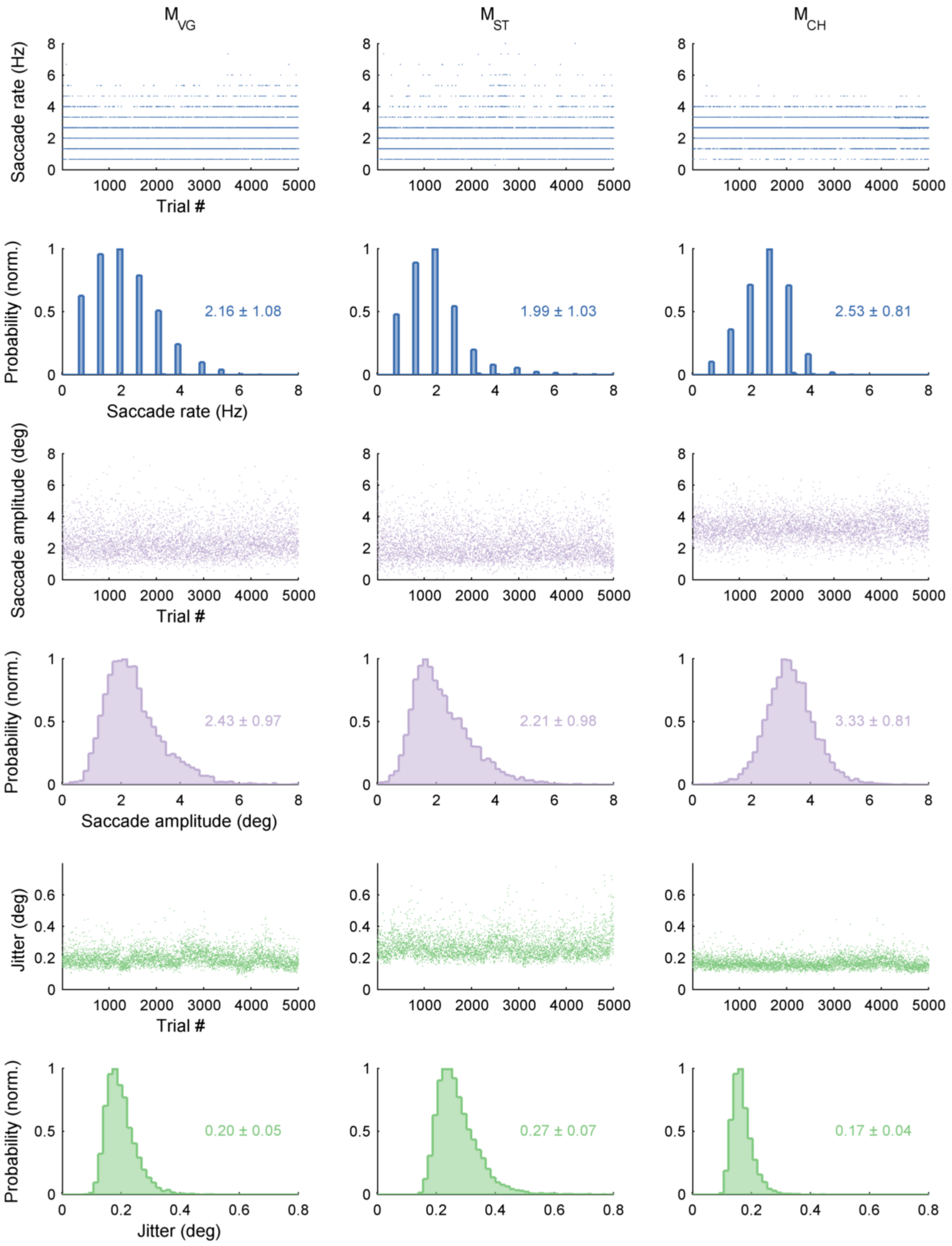

Supplementary Figure 4 | Distributions of cue phase eye movement properties. The values for each cue phase predictor for each of the last 5,000 trials, which were used in the GLM procedure (see also Figure 6), are plotted for each animal (columns). Predictors and units are: saccade rate, Hz; saccade amplitude, degrees; microsaccadic jitter, degrees, with colors following from Figure 6 of the main text. The histograms for the predictor values are plotted normalized to the maximum probability of occurrence. The mean and standard deviation are given for each distribution.

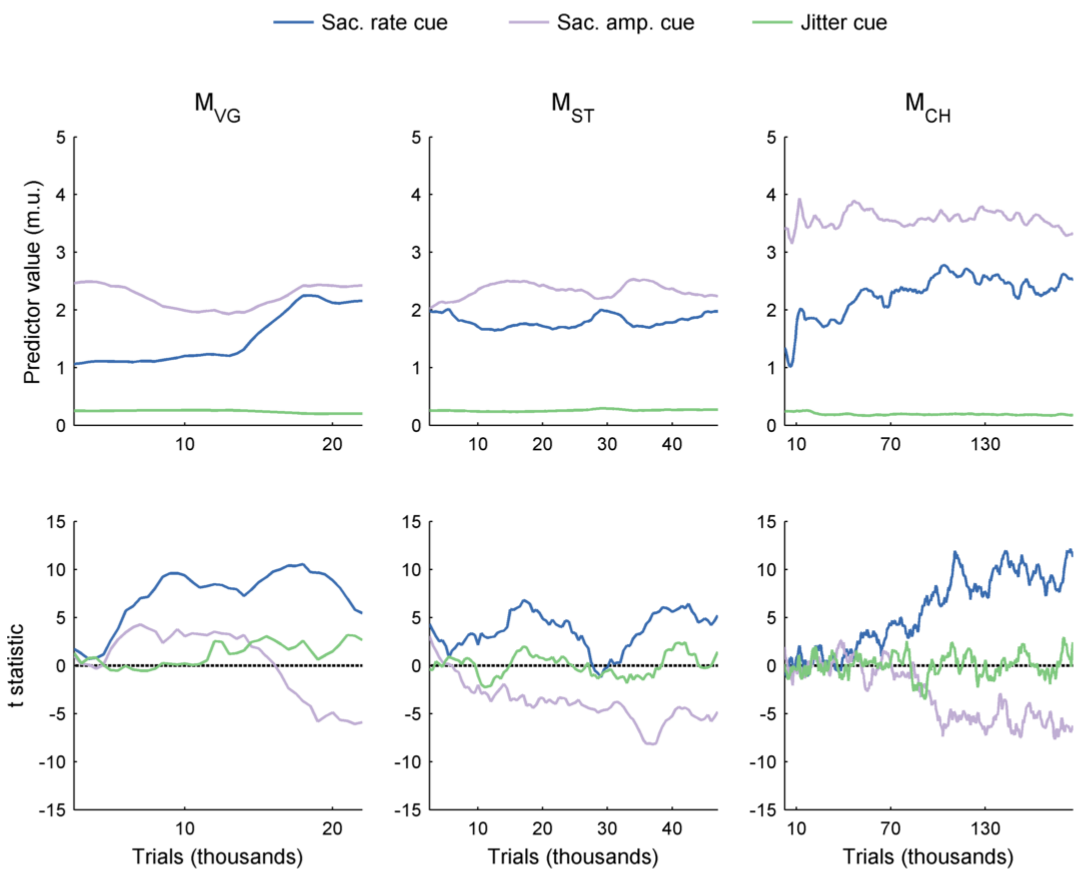

Supplementary Figure 5 | Time course of cue phase eye position predictors. *Top row:* The time course is shown for each predictor (m.u. mixed units, same as Supplementary Figure 4). *Bottom row:* The time course is shown for the t-statistic of the coefficient for each predictor in the GLM described in the main text (Figure 6). Sliding boxcar averages were taken to create the time courses in both panels (5,000 trials, 90% overlap; VA 1.3 to 1.9 and patterns p_1 to p_5). The values on the horizontal axis are the bin centers.

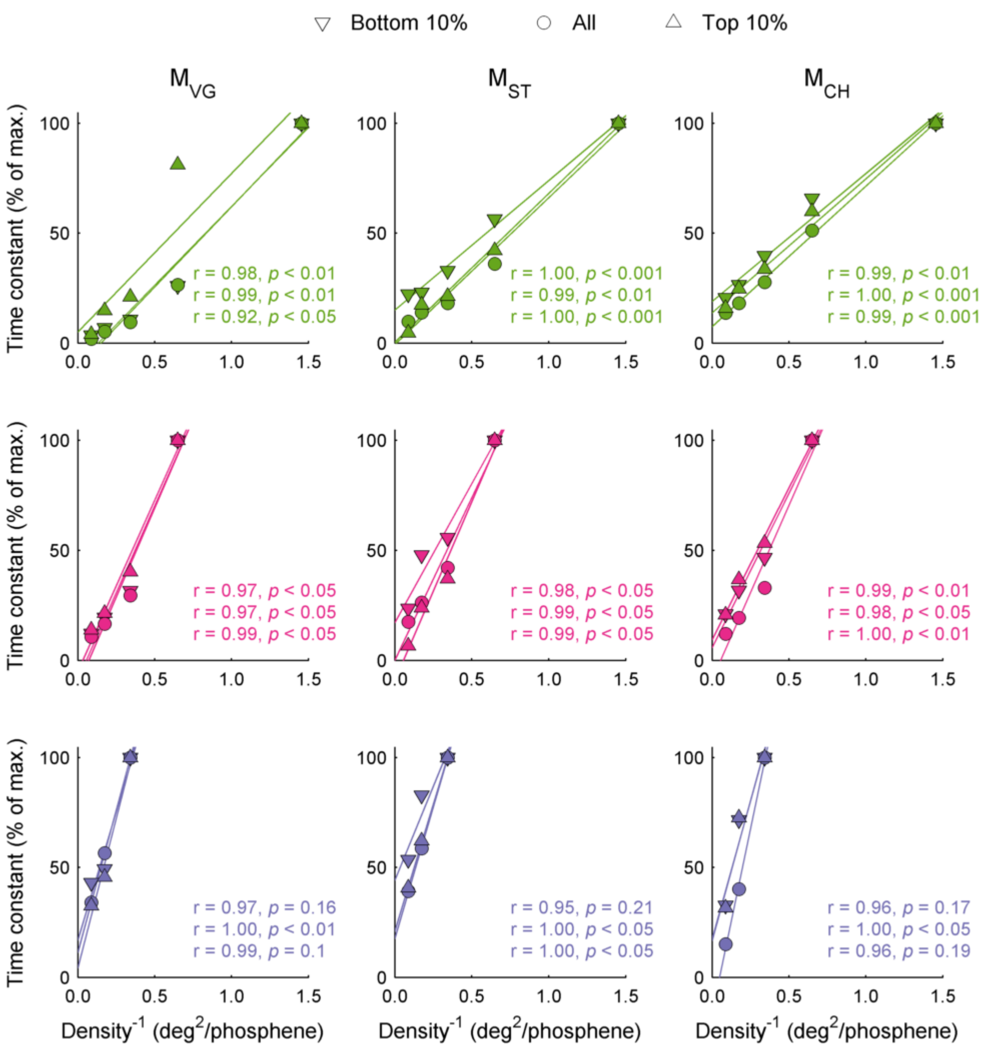

Supplementary Figure 6 | Learning time courses are invariant with respect to predictor values. The learning time constants are shown after performing calculations using subsets of trials when considering the effect of the following predictors: *K*, cue phase saccade rate, and cue phase saccade amplitude. Predictor values across all trials were sorted according to their effect on performance, either positive or negative, as identified through the GLM (see Figures 5, 6, and Supplementary Figures 2 through 5). If on any given trial a predictor value was in the top or best 10% of values, then the respective trial was included in the *Top 10%* stratum. Likewise, if on any given trial a predictor value was in the bottom or worst 10% of values then that trial was included in the *Bottom 10%* stratum. The results of the analysis from the Top 10% stratum are represented by upward-pointing triangles, the results from the Bottom 10% stratum are represented by downward-pointing triangles, and the results from all trials are represented by circles. *The learning time constants with subsets of trials have a relationship that is practically indistinguishable from the same relationship found when considering all trials.* The Pearson's product-moment correlation coefficient value and *p*-value are listed inside each panel in this order, going from top to bottom: Bottom 10%, All, Top 10%. The time constants are shown for each animal (columns) and for the pattern and VA level selections used in Figure 7 of the main text. Colors represent the VA level, as in Figure 7.

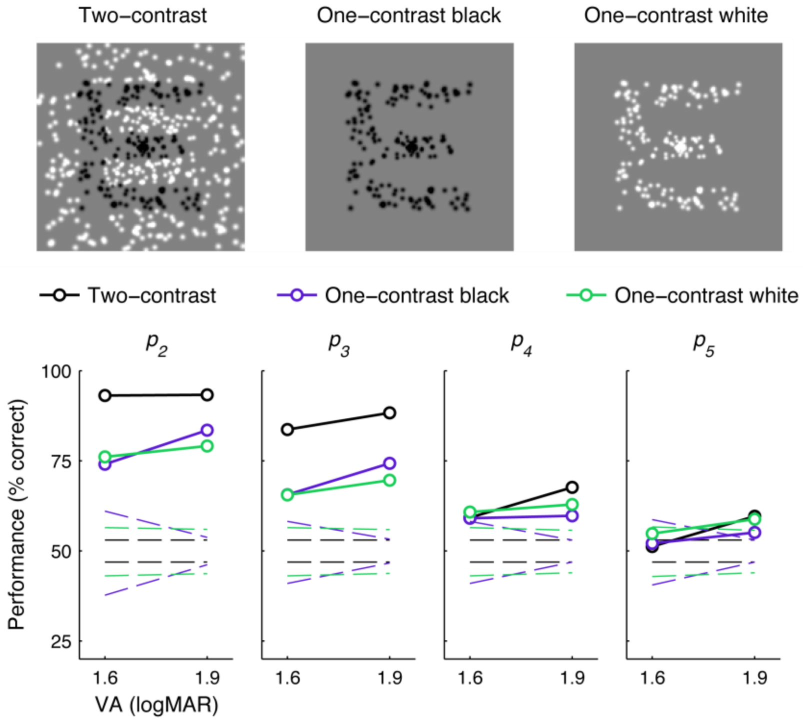

Supplementary Figure 7 | The dependence of performance upon contrast. Performance with the two-contrast phosphene view used for the primary results reported here was compared to performance with one-contrast phosphene views at the two highest VA levels (two largest font sizes). The two-contrast view is the standard view used for the main experiments that used black glyphs within a white square on a gray background, and thus both the glyph and contrasting field were represented. One animal was subsequently tested with one-contrast views that eliminated the white square, placing either a black or white glyph directly on the gray background. Example static phosphene view letterforms are shown at top for the three contrast cases with pattern p_2. On a block-by-block basis the letters were represented through either only black, or only white phosphenes. Performance was nearly the same for both one-contrast views, but one-contrast performance was lower than two-contrast performance, particularly at higher pattern densities. The difference at the two highest pattern densities could be due to increased local luminance contrast (black-white vs. black-gray or white-gray) and the additional spatial contrast information available with two luminance contrast levels whereby both positive and negative shape are consistently represented. The benefits of multiple contrast levels may be relatively weaker at lower pattern densities. Each data point represents the mean performance on up to the last 1,000 trials performed. The stippled lines correspond to a 95% confidence interval for the binomial test.

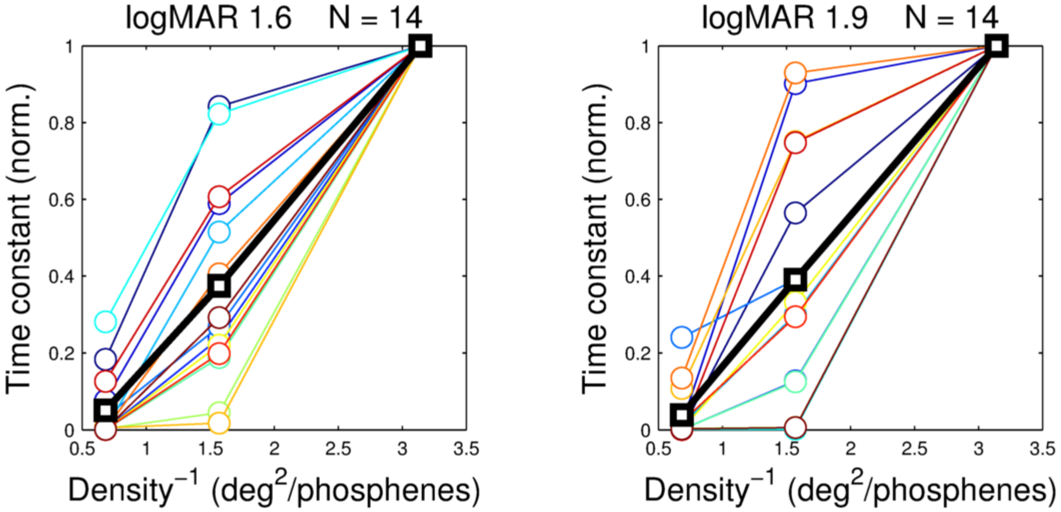

Supplementary Figure 8 | Learning time constants in humans. We examined performance data from 14 subjects tested with the experiments outlined in Bourkiza et al., 2013. Subjects and conditions were used where performance sufficiently evolved such that learning curves could be fit. We found that the learning time constants had the same relationship with inverse density as was found in non-human primates as shown here for two VA levels (compare to Figure 7b). Each color corresponds to a different subject. Learning was observed within the first and only testing session for each subject. The estimation error is therefore considerably higher in humans compared to non-human primates because there are far fewer trials per condition per human subject. The average across subjects shown by the heavy black line, however, agrees with the findings in non-human primates.

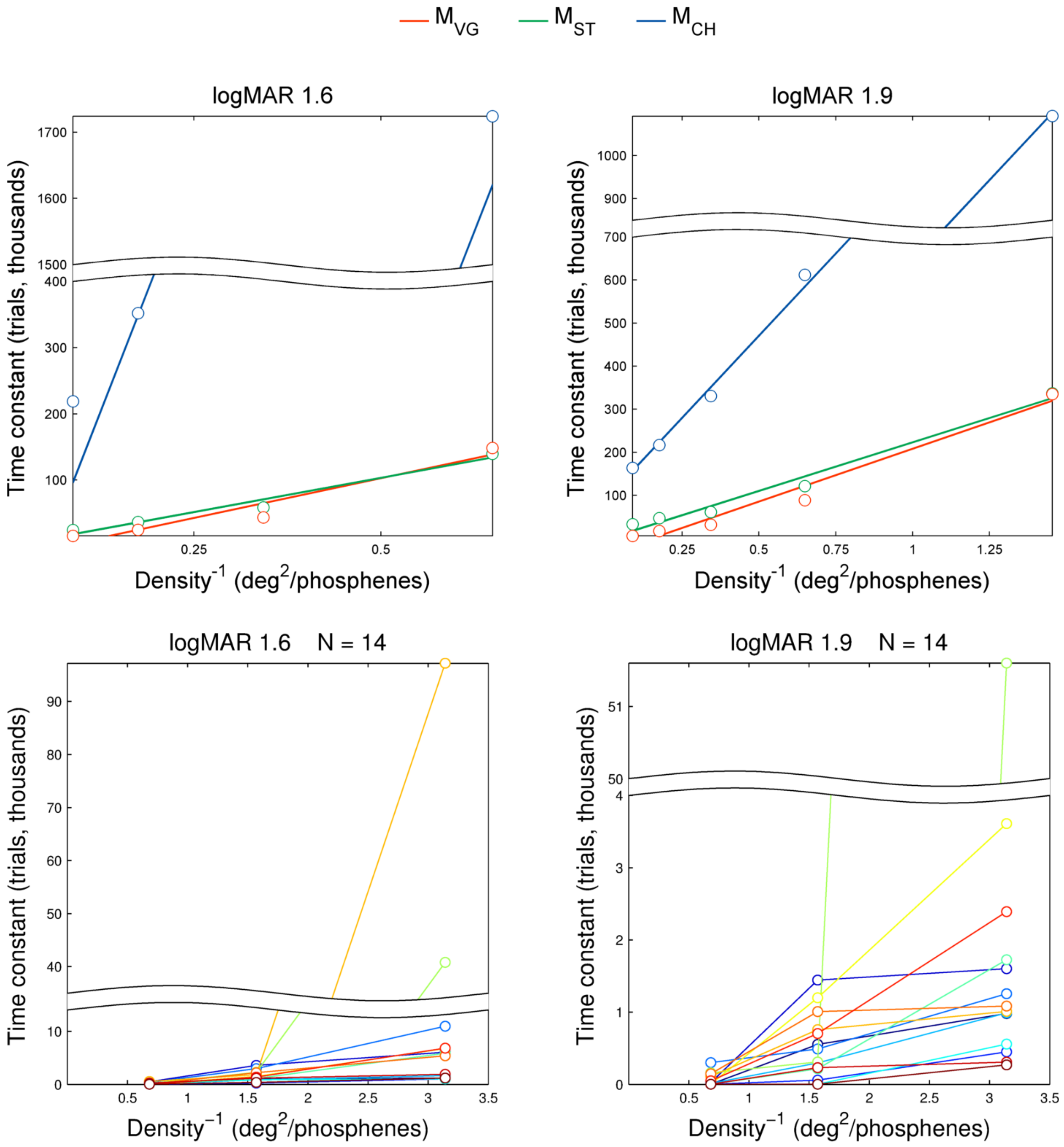

Supplementary Figure 9 | Comparison of learning time constants in non-human primates and humans. Learning time constants with best-fit lines for non-human primates, which were presented in normalized form in Figure 7, are shown in the top row. Learning time constants for humans, which were presented in normalized form in Supplementary Figure 8, are shown in the bottom row. Note that data points for human subjects are connected by line segments, not best-fit lines.

Supplementary References

1. Ferris, F. L. & Bailey, I. Standardizing the measurement of visual acuity for clinical research studies: Guidelines from the Eye Care Technology Forum. *Ophthalmology* **103,** 181–182 (1996).
2. Kaiser, P. K. Prospective evaluation of visual acuity assessment: a comparison of snellen versus ETDRS charts in clinical practice (An AOS Thesis). *Trans. Am. Ophthalmol. Soc.* **107,** 311–324 (2009).
3. Sloan, L. L., Rowland, W. M. & Altman, A. Comparison of three types of test target for the measurement of visual acuity. *Q Rev Ophthalmol* **8,** 4–16 (1952).
4. Pelli, D. G., Robson, J. G. & Wilkins, A. J. The design of a new letter chart for measuring contrast sensitivity. *Clin. Vis. Sci.* **2,** 187–199 (1988).
